# Measures of perceived mobility ability in community-dwelling older adults: a systematic review of psychometric properties

**DOI:** 10.1093/ageing/afad124

**Published:** 2023-10-30

**Authors:** Marla Beauchamp, Qiukui Hao, Ayse Kuspinar, Gésine Alder, Keitaro Makino, Mina Nouredanesh, Yunli Zhao, Christopher Mikton, Jotheeswaran Amuthavalli Thiyagarajan, Theresa Diaz, Parminder Raina

**Affiliations:** School of Rehabilitation Science, McMaster University, Hamilton, ON, Canada; McMaster Institute for Research on Aging, McMaster University, Hamilton, ON, Canada; School of Rehabilitation Science, McMaster University, Hamilton, ON, Canada; McMaster Institute for Research on Aging, McMaster University, Hamilton, ON, Canada; School of Rehabilitation Science, McMaster University, Hamilton, ON, Canada; McMaster Institute for Research on Aging, McMaster University, Hamilton, ON, Canada; McMaster Institute for Research on Aging, McMaster University, Hamilton, ON, Canada; McMaster Institute for Research on Aging, McMaster University, Hamilton, ON, Canada; Department of Preventive Gerontology, National Center for Geriatrics and Gerontology, Obu, Japan; School of Rehabilitation Science, McMaster University, Hamilton, ON, Canada; McMaster Institute for Research on Aging, McMaster University, Hamilton, ON, Canada; Department of Health Research Methods, Evidence, and Impact, McMaster University, Hamilton, ON, Canada; Demographic Change and Healthy Aging Unit, Social Determinants of Health, World Health Organization, Geneva, Switzerland; Ageing and Health Unit, Department of Maternal, Newborn, Child and Adolescent Health and Ageing, World Health Organization, Geneva, Switzerland; Department of Maternal, Newborn, Child and Adolescent Health and Ageing, World Health Organization, Geneva, Switzerland; McMaster Institute for Research on Aging, McMaster University, Hamilton, ON, Canada; Department of Health Research Methods, Evidence, and Impact, McMaster University, Hamilton, ON, Canada; Labarge Centre for Mobility in Aging, McMaster University, Hamilton, ON, Canada

**Keywords:** mobility, self-reported, perceived mobility, measurement properties, older people

## Abstract

**Objectives:**

The objective of this systematic review was to synthesise the psychometric properties of measures of perceived mobility ability and related frameworks used to define and operationalise mobility in community-dwelling older adults.

**Methods:**

We registered the review protocol with PROSPERO (CRD42022306689) and included studies that examined the psychometric properties of perceived mobility measures in community-dwelling older adults. Five databases were searched to identify potentially relevant primary studies. We qualitatively summarised psychometric property estimates and related operational frameworks. We conducted risk of bias and overall quality assessments, and meta-analyses when at least three studies were included for a particular outcome. The synthesised results were compared against the Consensus-based Standards for the Selection of Health Measurement Instruments criteria for good measurement properties.

**Results:**

A total of 36 studies and 17 measures were included in the review. The Late-Life Function and Disability Index: function component (LLFDI-FC), lower extremity functional scale (LEFS), Mobility Assessment Tool (MAT)-short form (MAT-SF) or MAT-Walking, and Perceived Driving Abilities (PDA) Scale were identified with three or more eligible studies. Most measures showed sufficient test–retest reliability (moderate or high), while the PDA scale showed insufficient reliability (low). Most measures had sufficient or inconsistent convergent validity (low or moderate) or known-groups validity (low or very low), but their predictive validity and responsiveness were insufficient or inconsistent (low or very low). Few studies used a conceptual model.

**Conclusion:**

The LLFDI-FC, LEFS, PDA and MAT-SF/Walking can be used in community-dwelling older adults by considering the summarised psychometric properties. No available comprehensive mobility measure was identified that covered all mobility domains.

## Key Points

- LLFDI-FC, LEFS, PDA and MAT-SF/Walking can be used in community-dwelling older adults by considering the summarised psychometric properties.
- There is no existing comprehensive mobility measure that covers all the mobility domains described by the World Health Organization.
- Further studies are needed on the responsiveness and predictive validity of self-report mobility measures.

## Introduction

The World Health Organization (WHO) has described healthy ageing as ‘the process of developing and maintaining the functional ability that enables wellbeing in older age’ [1]. Functional ability comprises the health-related attributes that enable people to be and to do what they value. To assess the impact of healthy ageing actions at the national, regional and global levels, WHO proposed functional ability as an indicator of healthy ageing.

In the recently released baseline report for the Decade of Healthy Ageing 2021–2030, WHO identified the ability to be mobile as being a critical indicator of the functional ability of older people across high-, middle- and low-income countries. According to the WHO report, mobility encompasses ‘movement in all its forms, whether powered by the body or a vehicle’ [2]. Mobility is critical for healthy ageing with respect to both physical and psychological well-being as it is a prerequisite for participation in most physical and social activities [3] and is a consistently strong predictor of changes in quality of life over time [4–6]. It is also a key element of frailty and sarcopenia assessments and provides helpful information for tailoring interventions [7].

Most previous reviews of psychometric properties of mobility measures have focused on performance-based measures (i.e. measures of locomotor capacity for mobility) or populations with specific conditions [8–11]. One (2016) review [12] summarised the measurement properties of 34 patient-reported mobility questionnaires in various adult populations and found that only 10 of them exclusively measured mobility activities. This review also noted that most of the existing patient-reported mobility measures (18/34) at that time were not based on the WHO International Classification of Functioning, Disability and Health (ICF) conceptual framework. This highlights that although many mobility frameworks have been proposed, there remains no gold standard for defining and operationalising mobility.

In light of the lack of conceptual clarity related to mobility measurement, in this WHO *Age and Ageing* special issue on measurements of healthy ageing, we proposed a unified framework for mobility measurement that builds on existing frameworks and evidence [13], but that is also in line with current terminology in the World Report on Ageing and Health [14]. Our framework outlined three distinct facets of mobility to consider for comprehensive mobility measurement in older people: (i) perceived mobility (i.e. self-reported ability or difficulty with task performance); (ii) actual mobility (i.e. frequency, duration or counts of mobility in daily life); (iii) locomotor capacity for mobility (i.e. performance on physical tests of mobility). This review focuses on the first category of mobility measurement: perceived mobility measures.

To our knowledge, there has been no previous systematic review of the psychometric properties of perceived mobility measures in community-dwelling older adults. The objective of this review was to synthesise the psychometric properties of available measures of perceived mobility in community-dwelling older adults and to summarise related frameworks used to define and operationalise mobility in the existing literature.

## Methods

### Mobility measures

This review focuses on self-report measures of perceived mobility for older community-dwelling adults. We registered the review protocol with PROSPERO (CRD42022306689). The WHO World Report on Ageing and Health describes the ability to be mobile as ‘movement in all its forms by the body (with or without an assistive device) and/or a vehicle’ and proposes the following sub-domains: getting up from a chair or moving from a bed to a chair, walking for leisure, exercising, completing daily tasks, driving a car and/or using public transportation [2, 15]. We used this definition of ability to be mobile to identify eligible measures. If a measure included both mobility- and non-mobility-related items, we only included the measure if 80% or more of the items were related to mobility or if it had a sub-domain solely related to the mobility construct.

As we were interested in measures that address older people’s perception of their ability to be mobile, i.e. what they say they can do [16], we included scales that asked ‘Are you able to. . .’, ‘Do you have any difficulty. . .’, ‘Do you require assistance’, ‘How difficult is it to. . .’ and so on. Measures that focused only on quantifying actual mobility levels (i.e. frequency or duration of an activity, calories expended, etc.) or solely on activities of daily living (e.g. continence, grooming) were excluded. Likewise, performance-based tests of locomotor capacity for mobility (e.g. gait speed) were also excluded and are covered in a separate review in this issue.

### Literature search and study identification

We used MEDLINE, EMBASE, CINAHL, PsycINFO and Ageline to conduct a comprehensive search to identify potentially eligible studies that reported psychometric properties of mobility measures up to March 2022. We included studies of community-dwelling older adults with at least 50% or more of participants aged 60 or over unless a separate analysis on participants aged 60 years and over was performed. The full search strategies are in Supplementary Appendix A.

### Study selection

We included studies with any design that focused on psychometric properties of perceived mobility measures among community-dwelling older adults with or without reference to a specific framework. Eligible studies met all the following criteria: (i) focused on at least one self-reported perceived mobility measure; (ii) reported on one or more of the following psychometric properties: reliability (including test–retest reliability, inter-rater reliability, intra-rater reliability measured by correlation coefficients), construct validity (including face validity, structural validity, cross-cultural validity, hypotheses-testing), criterion validity (concurrent validity, predictive validity), internal consistency (Cronbach’s alpha), measurement error (standard error of measurement (SEM)), responsiveness and interpretability measured by distribution or anchor-based methods; and (iii) included older adults (with a cut-off of 60 or over) living in the community. We excluded the following records: (i) only in abstract format, conference proceedings, editorials or commentaries; (ii) published in languages other than English; (iii) reviews.

Four reviewers independently manually screened 20% of titles and abstracts [17]. The screening orders were determined by Rayyan’s compute rating feature for systematic reviews. The compute rating was calculated by Rayyan based on previous decisions during the manual screening and default machine learning algorithms. Previous work has shown that this method has high specificity (99–100%) for excluding irrelevant citations [18]. Then, using Covidence, five reviewers independently performed full-text screening of all included records. Disagreements between reviewers in the full-text screening stage were resolved by discussion or, if needed, by consultation with a third reviewer.

### Data abstraction

One reviewer extracted data using a pre-designed data extraction sheet. All the extracted data were checked by a second reviewer. The following information was extracted: first author name; publication date; country(ies) and Low-to-Middle-High-Income status according to World Bank Data; study design; characteristics of the population (e.g. mean or median age, sample size, sex, rural or urban); mobility measure details (e.g. name of the measure, definition/framework/construct, sub-domains); psychometric property details (reliability, validity, consistency, measurement error, responsiveness and interpretability) and related frameworks. We followed COSMIN’s definition of responsiveness, which is ‘the ability of a measure to detect change over time’. In the included studies, different methods were used to define the minimal important difference (MID) or to test responsiveness. We used the reported results regardless of how the authors of individual studies have defined them and recognise that the method variability might result in different face values for MID and interpretations of responsiveness.

### Quality assessment of studies included

When at least three studies were included for an outcome measure, the quality of each eligible study was evaluated using the Consensus-based Standards for the Selection of Health Measurement Instruments (COSMIN) checklist [19]. The checklist includes 3 to 35 items for 10 psychometric properties in separate boxes and allows for rating the methodological quality as very good, adequate, doubtful and inadequate or not applicable. The methodological quality of the corresponding psychometric property was rated as the lowest rating [20]. Then, we evaluated the overall quality of evidence using the modified Grading of Recommendations, Assessment, Development and Evaluation (GRADE) [19, 21].

According to the COSMIN handbook for the systematic review of psychometric properties, we assessed the risk of bias, imprecision, inconsistency and indirectness in rating the overall quality of evidence [22]. We rated the overall quality of evidence as high to begin and adjusted the ratings based on the following existing issues. If there were any studies of doubtful or inadequate quality available, or there was only one study of adequate quality, we downgraded the evidence by one or two levels for risk of bias. For imprecision, we considered the sample size of related studies, and for inconsistency, we evaluated the proportions of studies that met pre-specified hypotheses for good measurement properties. If studies included only partial participants older than 60 years or from community settings, we downgraded the evidence for indirectness. Finally, the evidence was rated as high, moderate, low or very low. Additional details on the GRADE criteria can be found in Supplementary Appendix F.

### Synthesis of results

The major review findings were qualitatively summarised in tables, including psychometric property estimates, related operational frameworks and methods. We assessed the synthesised results against the revised COSMIN criteria [22] for good measurement properties to determine a rating of sufficient (+): at least 75% of hypotheses were met, insufficient (−): at least 75% of hypotheses were not met, inconsistent (±): mixed results (sufficient and insufficient <75%) or indeterminate (?): all results across eligible studies were indeterminant.

For test–retest reliabilities, meta-analyses were performed to pool available intraclass correlation coefficients (ICCs) after Fisher’s conversion based on the point estimate and sample size to stabilise the variances and approximate the ICC to be consistent with the normal distribution. We used DerSimonian and Laird random-effects models in the R ‘metacor’ package to conduct the meta-analysis [23]. The evidence was limited to performing the planned subgroup analyses according to age (<75 years versus >75 years), sex (male or female) and Low-to-Middle-High-Income status.

## Results

### Study selection and characteristics

**Figure 1:**
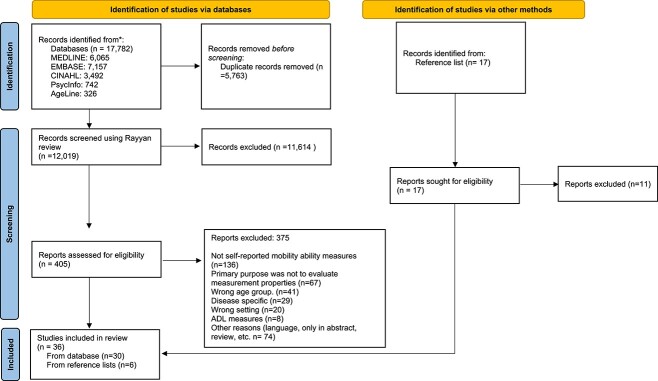
PRISMA flow diagram for selecting eligible studies. *Adapted from:* Page MJ, McKenzie JE, Bossuyt PM, Boutron I, Hoffmann TC, Mulrow CD, *et al*. The PRISMA 2020 statement: an updated guideline for reporting systematic reviews. *BMJ* 2021;372:n71. doi: 10.1136/bmj.n71.

The literature search yielded 12,019 citations after removal of duplicate records. Following the screening of title and abstracts, 11,614 citations were excluded and 405 potentially relevant studies from the database search were retrieved for full-text screening. Of these, 375 were excluded for various reasons, leaving 30 eligible studies. Seventeen relevant publications were retrieved from the hand search and reviewing of reference lists, and six proved eligible. Therefore, a total of 36 studies were included in this review [24–59] covering a total of 17 measures (see Supplementary Appendix B for characteristics of included measures). Figure 1 summarises the search and screening process for eligible studies.

### Perceived mobility measures

Nine of these studies reported the psychometric properties for the Late-Life Function and Disability Index: function component (LLFDI-FC) overall scales and subscales [24, 25, 31, 35, 41, 45, 46, 53, 55]; four studies reported psychometric properties for the lower extremity functional scale (LEFS) [47, 48, 56, 58]; five studies for the Mobility Assessment Tool (MAT)-short form (MAT-SF) [34, 39, 49, 51] or MAT-Walking [44]; and three studies for the Perceived Driving Abilities (PDA) Scale [27, 29, 42]; and 15 other studies reported on another 13 mobility scales or items [26, 28, 30, 32, 33, 36–38, 40, 43, 50, 52, 54, 57, 59] (Supplementary Appendix B). Most mobility measures did not mention related theoretical frameworks or models in their development. Frameworks or models mentioned included Nagi’s disablement model (LLFDI-FC), The International Classification of Functioning, Disability and Health (ICF) framework (Patient-Reported Outcomes Measurement Information System, PROMIS and Pepper Assessment Tool for Disability, PAT-D), Activity Space Model of Disability (self-reported measures of walking, running and lifting abilities) [40], and development of Mobility Limitation framework (Preclinical and manifest mobility limitation) [43]. All these frameworks focus on limitations in physical functioning.

The appendix includes a summary of identified measures (B), hypotheses for measures with good psychometric properties (C), characteristics of the 36 eligible studies (D), the GRADE assessment details (E) and detailed results tables (F–H).

1. 1 Late-Life Function and Disability Index: function component

Nine studies on the LLFDI-FC were identified in this review. Six of nine studies were conducted in the USA in English [24, 25, 35, 41, 46, 55]. Two studies were conducted in Israel (Hebrew version) [31, 45] and one study was conducted in Sweden using a Swedish version [53].

- Internal consistency and reliability

High-quality evidence from two studies [35, 53] demonstrated that LLFDI-FC overall score and subscales showed sufficient internal consistency: Cronbach alphas of 0.96–0.97 for the overall score, 0.86–0.94 for the upper extremity subscale, 0.94–0.96 for the basic lower extremity subscale and 0.95–0.96 for the advanced lower extremity subscale.

**Figure 2:**
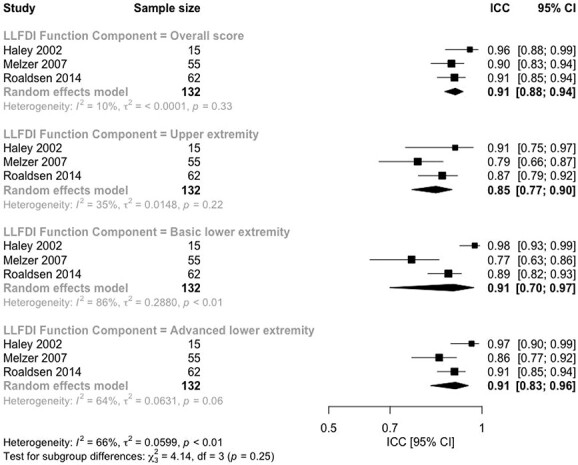
Test–retest reliability for Late-Life Function and Disability Index: function component.

High-quality evidence from three studies [35, 45, 53] demonstrated that LLFDI-FC overall score and subscales showed sufficient test–retest reliability: pooled ICC = 0.91; 95% confidence interval (CI): 0.88–0.94 for the overall scale, 0.85; 95% CI: 0.77–0.90 for the upper extremity subscale, 0.91; 95% CI: 0.70–0.97 for the basic lower extremity subscale, 0.91; 95% CI: 0.83–0.96 for the advanced lower extremity subscale (Figure 2, Table 1).

Moderate-quality evidence from three studies [24, 25, 53] reported inconsistent standard error of measurement (SEM) for the LLFDI-FC overall score (1.59 to 2.9), basic lower extremity (1.88 to 4.4) and advanced lower extremity (2.6 to 4.3). High-quality evidence showed insufficient SEM for the upper extremity subscale (4.2 to 5.1) (Table 3).

- Validity

Moderate-quality evidence from four studies [31, 41, 53, 55] supported the sufficient or inconsistent cross-sectional convergent validity for LLFDI-FC overall score (Table 2). The correlations between the LLFDI-FC and other health status, function or disability scales ranged from 0.52 to 0.83 [41, 53]. Adequate correlations were observed between LLFDI-FC overall scores and performance-based measures (absolute *r* = 0.55 to 0.69) [41, 45, 55]. There was moderate-quality evidence to demonstrate that the LLFDI-FC upper extremity and advanced lower extremity scales had sufficient or inconsistent convergent validity, while the quality of evidence was low for the basic lower extremity scale (Table 2).

Low-quality evidence from two studies [35, 45] showed that the LLFDI-FC overall score and subscales had sufficient known-groups validity (functional limitation as measured by the physical functioning subscale (PF-10) of the SF-36 or cane users) (Table 2). Low-quality evidence from two studies [24, 46] showed that the LLFDI-FC had insufficient predictive validity for adverse outcomes (falls, emergency department visits, hospitalisation, low self-reported health) (Table 2).

- Responsiveness

Low- or very low-quality evidence from two studies [24, 25] showed that the LLFDI-FC overall and advanced lower-extremity subscale had inconsistent responsiveness, while the upper extremity subscale and advanced lower-extremity subscale had insufficient responsiveness (Table 3).

- Change scores and interpretability

Four studies reported change scores for the LLFDI-FC [24, 25, 41, 53] with a minimal detectable change (MDC) with 95% confidence (MDC_95_) of 4.3 and MDC_90_ of 3.7 for the overall score, and an absolute and relative (%) smallest real difference of 8.0 (14%). The minimal clinically important difference was reported as 2 for the overall score, 4 for upper extremity and advanced lower-extremity subscales, and 3 for the basic lower-extremity subscale. Two studies [41, 53] noted that no floor or ceiling effects were observed for the LLFDI-FC.

There was a strong correction between the self-reported and interview-administered LLFDI-FC overall score (*r* = 0.95) [41]. One study [31] reported that the basic lower extremity showed acceptable reliability between self-reported scores and clinician assessment scores with an ICC of 0.83 (95% CI: 0.69 to 0.91), while the upper extremity subscale showed poor reliability with an ICC of 0.3 (95% CI: −0.18 to 0.62).

1. 2 Lower extremity functional scale

Four studies on the LEFS were identified in this review [47, 48, 56, 58]. Three studies were conducted in English in the USA [58], Canada [56] and Australia [48], and one study [47] was conducted in Slovenia using a Slovenian version.

- Internal consistency and reliability

Very low-quality evidence from one study [47] reported that the LEFS had sufficient internal consistency with a Cronbach’s alpha of 0.99 (Table 1). Moderate-quality evidence from three studies [47, 48, 58] showed that the LEFS had sufficient test–retest reliability with a pooled ICC of 0.97 (Table 1) and two studies [47, 48] reported sufficient SEM (0.97 and 3.6).

- Validity

**Table 1:** Self-reported mobility measures review: Reliability

**Reliability**	**# of tests/hypotheses**	**Summary or pooled result**	**Overall rating**	**Quality of evidence**
LLFDI-FC: Overall score	3	100% of hypotheses were confirmed; *see forest plot overall ICC = 0.91 (0.88–0.94)	Sufficient (+)	High
LLFDI-FC: upper extremity	3	100% of hypotheses were confirmed; ICC = 0.85 (0.77–0.90)	Sufficient (+)	High
LLFDI—basic L/E	3	100% of hypotheses were confirmed; ICC = 0.91 (0.70–0.97)	Sufficient (+)	High
LLFDI—advanced L/E	3	100% of hypotheses were confirmed; ICC = 0.91 (0.83–0.96)	Sufficient (+)	High
LEFS	3	100% of hypotheses were confirmed; ICC = 0.97 (0.91–0.99)	Sufficient (+)	Moderate^a^
PDA current	1	0% of hypotheses were confirmed; ICC = 0.65	Insufficient (±)	Low^b^
PDA changes	1	0% of hypotheses were confirmed; ICC = 0.66	Insufficient (±)	Low^b^
MAT-SF	3	100% of hypotheses were confirmed; ICC = 0.91 (0.81–0.96)	Sufficient (+)	High
MAT-Walking	1+ for each sub-score	100% of hypotheses were confirmed; ICC = 0.85–0.89	Sufficient (+)	Low^b^

^a^Due to serious indirectness; ^b^due to very serious imprecision.

Low-quality evidence showed that the LEFS had inconsistent convergent validity (Table 2). Two studies [48, 58] reported that LEFS had moderate correlations with self-report measures of physical function (*r* = 0.60 to 0.78) and performance-based measures (*r* = 0.40 to 0.69). Very low-quality evidence from one study [56] supports LEFS had sufficient known-groups validity. There were no studies on predictive validity of the LEFS.

- Change scores and interpretability

**Table 2:** Self-reported mobility measures review: Validity

**Convergent validity**	**# of tests/hypotheses**	**Summary or pooled result**	**Overall rating**	**Quality of evidence**
				
LLFDI-FC: overall score	16	100% of hypotheses were confirmed	Sufficient (+)	Moderate Due to serious indirectness
LLFDI-FC: upper extremity	8	75% of hypotheses were confirmed	Sufficient (+); inconsistent	Moderate Due to serious indirectness
LLFDI—basic L/E	8	87.5% of hypotheses were confirmed	Sufficient (+); inconsistent	Low Due to serious inconsistency and serious indirectness
LLFDI—advanced L/E	6	66.7% of hypotheses were confirmed	Inconsistent (±)	Moderate Due to serious inconsistency
LEFS	13	69% of hypotheses were confirmed	Inconsistent (±)	Low Due to serious inconsistency and serious indirectness
PDA current	6	50% of hypotheses were confirmed	Inconsistent (±)	Moderate Due to serious inconsistency
PDA changes	6	50% of hypotheses were confirmed	Inconsistent (±)	Moderate Due to serious inconsistency
MAT-SF	15	60% of hypotheses were confirmed	Inconsistent (±)	Moderate Due to serious inconsistency
MAT-Walking	4	50% of hypotheses were confirmed	Sufficient (+) for total walking distance; inconsistent (±) for walking speed	Moderate Due to serious inconsistency
**Known-groups validity**	**Number of tests**	**Summary or pooled result**	**Overall rating**	**Quality of evidence**
LLFDI-FC: overall score	2	100% of hypotheses were confirmed	Sufficient (+)	Low Due to very serious risk of bias
LLFDI-FC: upper extremity	2	100% of hypotheses were confirmed	Sufficient (+)	Low Due to very serious risk of bias
LLFDI—basic L/E	2	100% of hypotheses were confirmed	Sufficient (+)	Low Due to very serious risk of bias
LLFDI—advanced L/E	2	100% of hypotheses were confirmed	Sufficient (+)	Low Due to very serious risk of bias
LEFS	1	100% of hypotheses were confirmed	Sufficient (+)	Very low Due to imprecision and very serious risk of bias
MAT-SF	3	100% of hypotheses were confirmed	Sufficient (+)	Low Due to very serious risk of bias
**Predictive validity**	**# of tests/hypotheses**	**Summary or pooled result**	**Overall rating**	**Quality of evidence**
LLFDI-FC: overall score	5	0% of hypotheses were confirmed	Insufficient (−)	Low Due to very serious risk of bias
LLFDI—basic L/E	5	0% of hypotheses were confirmed	Insufficient (−)	Low Due to very serious risk of bias
LLFDI—advanced L/E	5	0% of hypotheses were confirmed	Insufficient (−)	Low Due to very serious risk of bias
**Structural validity**	**# of tests/hypotheses**	**Summary or pooled result**	**Overall rating**	**Quality of evidence**
MAT-SF	1	100% of hypotheses were confirmed	Sufficient (+)	Low Due to very serious risk of bias

**Table 3:** Self-reported mobility measures review: Responsiveness

**Responsiveness**	**Number of tests**	**Summary or pooled result**	**Overall rating**	**Quality of evidence**
LLFDI-FC: overall score	2	50% of hypotheses were confirmed; score changes correlated with GRC (Rho = 0.3)	Inconsistent (±)	Very low Due to very serious risk of bias and serious inconsistency
LLFDI-FC: upper extremity	1	0% of hypotheses were confirmed	Insufficient (−)	Low Due to very serious risk of bias
LLFDI—basic L/E	2	0% of hypotheses were confirmed	Insufficient (−)	Low Due to very serious risk of bias
LLFDI—advanced L/E	2	50% of hypotheses were confirmed; score changes correlated with GRC (Rho = 0.3)	Inconsistent (−)	Very low Due to very serious risk of bias and serious inconsistency
MAT-Walking	1	100% of hypotheses were confirmed (total and fast walking distance showed larger effects than usual walking distance)	Sufficient (+)	Low Due to very serious risk of bias

One study [58] revealed that LEFS (change/SD = 1.2) is more sensitive to change after physical therapy than other function or balance measures (change/SD = 0.55 to 1.1). An MDC (not reported the confidence level) in healthy older adults of 2.69 was reported in one study [47] and an MDC_95_ of 9.9 (upper 95% CI = 12.5) was reported for older adults with hip osteoarthritis [48]. One study [48] noted that floor or ceiling effects were not observed for the LEFS.

1. 3 Perceived Driving Abilities Scale

Three studies included in this review assessed the PDA Scale and all were administered in Canada in the English language [27, 29, 42].

- Internal consistency and reliability

High-quality evidence from two studies [27, 42] showed that the PDA had sufficient internal consistency with Cronbach’s alpha of 0.92–0.94 for current abilities and 0.77–0.87 for perceived changes in driving ability (Table 1). Low quality of evidence (Table 1) from one study [27] showed insufficient test–retest reliabilities for PDA current (ICC = 0.65) and PDA change (ICC = 0.66).

- Validity

Moderate-quality evidence from two studies [27, 42] showed inconsistent convergent validity for the PDA (Table 2). The PDA current scale correlated with driving frequency, comfort and avoidance scales (*r* = 0.46 to 0.59) [27, 42], and actual driving behaviour (distance, duration and other measures) (*r* = 0.13 to 0.39) [27]. The PDA change scale also correlated with driving comfort scales (*r* = 0.43 to 0.49) [42], and driving frequency (*r* = −0.13) and avoidance (*r* = 0.21) scales [27].

Chen *et al*. (2021) reported that the accuracy of the PDA was 28.7%, with 52.8% of participants overestimating their ability and 18.5% of participants underestimating their ability [29]. There were no data on predictive validity.

1. 4 Mobility Assessment Tool: MAT-SF and MAT-Walking

Five studies reporting on the MAT were included in this review [34, 39, 44, 49, 51]. Four of them were conducted in the USA (English) [39, 44, 49, 51] and one study [34] was conducted in Brazil (Portuguese) and Colombia (Spanish).

- Internal consistency and reliability

High-quality evidence from three cohorts in two studies [34, 49] showed that MAT-SF had sufficient test–retest reliability with a pooled ICC of 0.91, 95% CI: 0.81 to 0.96 (Table 1). Low-quality evidence from one study [44] showed that MAT-Walking sub-score had sufficient test–retest reliability with ICCs of 0.85 for both usual-pace and fast-pace walking distance and 0.89 for total walking distance.

- Validity

Low-quality evidence from one study [51] showed the MAT-SF had sufficient structural validity with acceptable model fit for the overall unidimensional and goodness of fit for item level (Table 2). Moderate evidence from three studies [34, 49, 51] showed the MAT-SF had sufficient but inconsistent convergent validity (Table 2). The MAT-SF correlated with the Pepper Assessment Tool for Disability subscales [49] (*r* = 0.44 to 0.60). Low-quality evidence from two studies [49, 51] showed that the MAT-SF had sufficient known-groups validity (chronic disease or failure to complete the 400-m walk test) (Table 2).

Moderate evidence from one study [44] showed the MAT-SF had sufficient but inconsistent convergent validity: the MAT-Walking was related to speed data using objective walking speed (*r* = 0.36 and 0.45) and other physical activity measures (*r* = 0.66 and 0.65) [44]. No data were available for predictive validity.

- Responsiveness

Low-quality evidence from one study [44] showed that MAT-Walking had sufficient responsiveness (treatment effect: *η*^2^ = 0.18 to 0.38, *P* < 0.001) after a 6-month intervention [44].

- Interpretability

The MAT-SF did not show any floor or ceiling effects among older adults aged 70–89 years [51]. Proxy-reported mobility assessment using the MAT-SF also showed good reliability (ICC = 0.81) and the patient-reported MAT-SF showed a high correlation with proxy-reported MAT-SF (*r* = 0.81) [39].

1. 5 Other mobility measures

The Questionnaire Rising and Sitting down (QR&S) items are predominantly loaded on a single component and have good internal consistency (Cronbach’s alpha = 0.96) [54]. De Laat *et al*. [30] reported on QR&S test–retest reliability (ICC = 0.83, *n* = 22), with a relative SEM of 6.7% and the smallest detectable difference of 18.6%. The same study also demonstrated that the QR&S had good construct validity.

The PROMIS physical function may also be used to assess perceived mobility. The 10-item simulated computerised adaptive testing was better than static versions in information content and reliability in PROMIS [33], and the PROMIS *t*-score correlated with the modified physical performance test overall scores (*r* = 0.59) and component scores (*r* = −0.28 to 0.64) [37]. Supplementary Appendix H summarises the reported psychometric properties for other self-reported mobility measures.

## Discussion

This systematic review summarised the psychometric properties of self-report measures that focus on community-dwelling older adults’ perceived mobility ability. We found several measures, including the LLFDI-FC, LEFS, PDA, MAT-SF/Walking and others, that reflected the concept of mobility as described by the WHO World Report on Ageing and Health, and that have been evaluated in community-dwelling older adults. Our findings on their psychometric properties can be used by researchers and clinicians to inform the selection of the most appropriate measures for assessing mobility in community-dwelling older adults.

Previous systematic reviews on mobility measures were conducted in different populations than our current review [12] or they focused only on one specific measure (such as the LLFDI [60] or LEFS [61]), or type of assessment [62]. This review included a broad range of outcome measures based on the WHO concept of ‘ability to be mobile’ and summarised measures that were valid among community-dwelling older adults. Previous summaries of the psychometric properties of the LLFDI and LEFS in other reviews [60, 61] are consistent with our main findings; however, in this review, we also assessed the synthesised results using COSMIN criteria for good measurement properties and GRADE for the overall quality of the evidence. Although the LLFDI-FC, MAT and LEFS were the most extensively evaluated measures in our review and had sufficient convergent validity, our results highlight an important gap with respect to predictive validity. The LLFDI-FC was the only measure with any evidence of its predictive validity, and reported AUCs did not reach COSMIN criteria (AUC of 0.7) (low-quality evidence). However, we acknowledge that the requirement of an AUC of 0.7 or higher for predictive ability, stipulated by the COSMIN criteria, may not be appropriate for mobility measurements and non-diagnostic work in observational studies in the community due to the complex contextual factors involved in mobility measurements. More work is necessary to determine the most appropriate criteria for evaluating the predictive ability of multifaceted mobility measures. Given the established role of mobility measurement for screening and risk stratification, there is an urgent need for longitudinal studies explicitly designed to evaluate the predictive validity of these mobility measures.

Most measures of perceived mobility in this review demonstrated sufficient internal consistency and reliability; however, the evidence was low and limited regarding their responsiveness. Responsiveness is important to consider in studies with the purpose of assessing the effectiveness of interventions in clinical trials [63]. Providing higher quality evidence on responsiveness for mobility measures will require future studies using clear hypotheses for treatment effect size and robust designs.

Some technology-dependent mobility measures emerged in this review (e.g. MAT-SF, PROMIS physical function), which had better internal consistency, reliability and convergent validity than the traditional questionnaire format, but may require more resources to administer [33]. Future well-designed studies are needed to directly compare psychometric properties between traditional and technology-based measures of perceived mobility.

It was notable that few measures of mobility in this review cited using a conceptual framework, model or operational definition. Of the frameworks mentioned, Nagi’s disablement model and the ICF were the most common. However, these frameworks are not specific to mobility and do not distinguish among different aspects of mobility that can impact measurement properties. To advance the science of mobility measurement as an indicator of healthy ageing, in this issue of *Age and Ageing*, we proposed a new framework that outlines three facets of mobility measures [13]. Future studies may benefit from using our unified mobility measurement framework alongside the current WHO descriptions of relevant functional ability domains [14] to develop and test conceptually sound and comprehensive mobility measures.

This systematic review had several strengths. We did not restrict mobility to movement across different places, specific conditions or rehabilitation settings. We used a broad search that included both physical function and transportation measures. We closely followed the COSMIN methodology for systematic reviews of patient-reported outcome measures [22] that included developing the search, rating the risk of bias, assessing the overall quality of evidence and synthesising results based on explicit criteria.

Nonetheless, our review has some important limitations. First, there is no medical subject heading in MEDLINE for mobility that is consistent with our conceptualisation. To identify all potentially relevant mobility measures, we used broad search strategies that yielded an unmanageable number of records for manual screening and necessitated the use of machine learning approaches. We also restricted our search to older adults and self-reported outcome measures and we did not search the grey literature. Therefore, we may have missed some relevant studies or outcome measures. Second, in line with COSMIN guidelines, we only included studies that examined psychometric properties as one of their main purposes, which may miss relevant information, especially for assessment of predictive validity and responsiveness. Third, we did not assess the impact of publication bias on the overall quality of evidence due to a lack of guidance on this issue, but publication bias may have substantial influences on psychometric properties. Fourth, we also did not assess the content validity of the included measures, which was out of the scope of this review, and the limited evidence did not allow us to conduct our planned subgroup analyses by age, sex and Low-to-Middle-High-Income status. Finally, we did not find any comprehensive mobility measures that covered all mobility domains outlined by the WHO. Future studies are likely needed to develop comprehensive mobility measures that reflect all the sub-domains of mobility in one measure.

Although further prospective studies are required to develop a comprehensive conceptually relevant self-perceived mobility measure with all acceptable psychometric properties, in the meantime, a simple mobility indicator may be useful to advance the WHO decade of healthy ageing and influence health-care policies, particularly in low- and middle-income countries. It is important to note that in low- and middle-income countries, where access to personal vehicles may be limited, other simple or basic mobility devices beyond cars may be more relevant. Therefore, when assessing the transportation domain of mobility measures in low- and middle-income countries, one needs to consider the availability of mobility devices, such as personal cars, public buses, canes, rollators, railings and other small mobility vehicles for shopping.

In conclusion, we summarised the psychometric properties of self-report measures that focus on perceived mobility ability in community-dwelling older adults. The LLFDI-FC, LEFS, PDA and MAT-SF/Walking can be used to assess perceived mobility in community-dwelling older adults; however, further data are needed on their responsiveness and predictive validity.

## Disclaimer

The authors alone are responsible for the views expressed in this article and they do not necessarily represent the views, decisions or policies of the institutions with which they are affiliated.

## Supplementary Material

aa-23-0436-File002_afad124

## Data Availability

This paper’s underlying data are available in the article and its supplementary data files.
