# Supplementary material for: Measures of perceived mobility ability in community-dwelling older adults: a systematic review of psychometric properties
Source: Age Ageing. 2023 Oct 30;52(Suppl 4):iv100–11. doi: 10.1093/ageing/afad124 (PMC10615037; doi:10.1093/ageing/afad124)
Supplement: aa-23-0436-File002_afad124 [file aa-23-0436-file002_afad124.docx]

World Health Organization: *Measurement of Healthy Ageing.*

**Measures of perceived mobility ability in community-dwelling older adults: A systematic review of psychometric properties**

SUPPLEMENTARY DATA

[Appendix A. Search strategies and numbers of citations identified 2](#_Toc129151823)

[Appendix B. Characteristics of Self-report Perceived Mobility Measures included in the Review 18](#_Toc129151824)

[Appendix C. Criteria for good measurement properties and related hypothesis 21](#_Toc129151825)

[Appendix D. Characteristics of the 36 eligible studies 24](#_Toc129151826)

[Appendix E. GRADE assessment details 32](#_Toc129151827)

[Appendix F. GRADE Criteria for the review 41](#_Toc129151828)

[Appendix G. Summary of finding tables for internal consistency, measurement error 43](#_Toc129151829)

[Appendix H. Reported psychometric property details 47](#_Toc129151830)

## Appendix A. Search strategies and numbers of citations identified

**OVID Medline Epub Ahead of Print, In-Process & Other Non-Indexed Citations, Ovid MEDLINE(R) Daily and Ovid MEDLINE(R) 1946 to Present**

1 (instrumentation or methods).fs. 4454551

2 (Validation Studies or Comparative Study).pt. 1910984

3 exp Psychometrics/ 83850

4 psychometr*.ti,ab. 54470

5 (clinimetr* or clinometr*).tw. 1307

6 exp "Outcome Assessment (Health Care)"/ 1275317

7 outcome assessment.ti,ab. 4489

8 outcome measure*.tw. 255322

9 exp Observer Variation/ 44526

10 observer variation.ti,ab. 1153

11 exp Health Status Indicators/ 336163

12 exp "Reproducibility of Results"/ 444220

13 reproducib*.ti,ab. 177915

14 exp Discriminant Analysis/ 11641

15 (reliab* or unreliab* or valid* or coefficient or homogeneity or homogeneous or "internal consistency").ti,ab. 1635355

16 (cronbach* and (alpha or alphas)).ti,ab. 27848

17 (item and (correlation* or selection* or reduction*)).ti,ab. 26698

18 (agreement or precision or imprecision or "precise values" or test-retest).ti,ab. 481621

19 (test and retest).ti,ab. 31415

20 (reliab* and (test or retest)).ti,ab. 103390

21 (stability or interrater or inter-rater or intrarater or intra-rater or intertester or inter-tester or intratester or intra-tester or interobserver or inter-observer or intraobserver or intraobserver or intertechnician or inter-technician or intratechnician or intra-technician or interexaminer or inter-examiner or intraexaminer or intra-examiner or interassay or interassay or intraassay or intra-assay or interindividual or inter-individual or intraindividual or intra-individual or interparticipant or inter-participant or intraparticipant or intra-participant or kappa or kappa's or kappas or repeatab*).ti,ab. 713994

22 ((replicab* or repeated) and (measure or measures or findings or result or results or test or tests)).ti,ab. 219633

23 (generaliza* or generalisa* or concordance).ti,ab. 106798

24 (intraclass and correlation*).ti,ab. 30346

25 (discriminative or "known group" or factor analysis or factor analyses or dimension* or subscale*).ti,ab. 755788

26 (multitrait and scaling and (analysis or analyses)).ti,ab. 145

27 (item discriminant or interscale correlation* or error or errors or "individual variability").ti,ab. 353553

28 (variability and (analysis or values)).ti,ab. 113085

29 (uncertainty and (measurement or measuring)).ti,ab. 9200

30 ("standard error of measurement" or sensitiv* or responsive*).ti,ab. 1755432

31 ((minimal or minimally or clinical or clinically) and (important or significant or detectable) and (change or difference)).ti,ab. 275852

32 (small* and (real or detectable) and (change or difference)).ti,ab. 8600

33 (meaningful change or "ceiling effect" or "floor effect" or "Item response model" or IRT or Rasch or "Differential item functioning" or DIF or "computer adaptive testing" or "item bank" or "cross-cultural equivalence").ti,ab. 15940

34 or/1-33 10604656

35 ((mobil* adj3 questionnaire) or (mobil* adj3 instrument) or (mobil* adj3 scale) or (mobil* adj3 index) or (mobil* adj3 score) or (mobil* adj3 measure) or (mobil* adj3 tool)).mp. or exp mobility limitation/ 9538

36 ((move* adj3 questionnaire) or (move* adj3 instrument) or (move* adj3 scale) or (move* adj3 index) or (move* adj3 score) or (move* adj3 measure) or (move* adj3 tool)).mp. [mp=title, abstract, original title, name of substance word, subject heading word, floating sub-heading word, keyword heading word, organism supplementary concept word, protocol supplementary concept word, rare disease supplementary concept word, unique identifier, synonyms] 5457

37 ((abilit* adj3 questionnaire) or (abilit* adj3 instrument) or (abilit* adj3 scale) or (abilit* adj3 index) or (abilit* adj3 score) or (abilit* adj3 measure) or (abilit* adj3 tool)).mp. [mp=title, abstract, original title, name of substance word, subject heading word, floating sub-heading word, keyword heading word, organism supplementary concept word, protocol supplementary concept word, rare disease supplementary concept word, unique identifier, synonyms] 11233

38 ((walk* adj3 questionnaire) or (walk* adj3 instrument) or (walk* adj3 scale) or (walk* adj3 index) or (walk* adj3 score) or (walk* adj3 measure) or (walk* adj3 tool)).mp. [mp=title, abstract, original title, name of substance word, subject heading word, floating sub-heading word, keyword heading word, organism supplementary concept word, protocol supplementary concept word, rare disease supplementary concept word, unique identifier, synonyms] 3475

39 ((limitation? adj3 questionnaire) or (limitation? adj3 instrument) or (limitation? adj3 scale) or (limitation? adj3 index) or (limitation? adj3 score) or (limitation? adj3 measure) or (limitation? adj3 tool)).mp. [mp=title, abstract, original title, name of substance word, subject heading word, floating sub-heading word, keyword heading word, organism supplementary concept word, protocol supplementary concept word, rare disease supplementary concept word, unique identifier, synonyms] 2402

40 ((function* adj3 questionnaire) or (function* adj3 instrument) or (function* adj3 scale) or (function* adj3 index) or (function* adj3 score) or (function* adj3 measure) or (function* adj3 tool)).mp. [mp=title, abstract, original title, name of substance word, subject heading word, floating sub-heading word, keyword heading word, organism supplementary concept word, protocol supplementary concept word, rare disease supplementary concept word, unique identifier, synonyms] 66626

41 ((transfer* adj3 questionnaire) or (transfer* adj3 instrument) or (transfer* adj3 scale) or (transfer* adj3 index) or (transfer* adj3 score) or (transfer* adj3 measure) or (transfer* adj3 tool)).mp. [mp=title, abstract, original title, name of substance word, subject heading word, floating sub-heading word, keyword heading word, organism supplementary concept word, protocol supplementary concept word, rare disease supplementary concept word, unique identifier, synonyms] 2864

42 ((ambulat* adj3 questionnaire) or (ambulat* adj3 instrument) or (ambulat* adj3 scale) or (ambulat* adj3 index) or (ambulat* adj3 score) or (ambulat* adj3 measure) or (ambulat* adj3 tool)).mp. [mp=title, abstract, original title, name of substance word, subject heading word, floating sub-heading word, keyword heading word, organism supplementary concept word, protocol supplementary concept word, rare disease supplementary concept word, unique identifier, synonyms] 1439

43 ((activit* adj3 questionnaire) or (activit* adj3 instrument) or (activit* adj3 scale) or (activit* adj3 index) or (activit* adj3 score) or (activit* adj3 measure) or (activit* adj3 tool)).mp. [mp=title, abstract, original title, name of substance word, subject heading word, floating sub-heading word, keyword heading word, organism supplementary concept word, protocol supplementary concept word, rare disease supplementary concept word, unique identifier, synonyms] 55530

44 ((disabilit* adj3 questionnaire) or (disabilit* adj3 instrument) or (disabilit* adj3 scale) or (disabilit* adj3 index) or (disabilit* adj3 score) or (disabilit* adj3 measure) or (disabilit* adj3 tool)).mp. [mp=title, abstract, original title, name of substance word, subject heading word, floating sub-heading word, keyword heading word, organism supplementary concept word, protocol supplementary concept word, rare disease supplementary concept word, unique identifier, synonyms] 29122

45 ((exercise adj3 questionnaire) or (exercise adj3 instrument) or (exercise adj3 scale) or (exercise adj3 index) or (exercise adj3 score) or (exercise adj3 measure) or (exercise adj3 tool)).mp. [mp=title, abstract, original title, name of substance word, subject heading word, floating sub-heading word, keyword heading word, organism supplementary concept word, protocol supplementary concept word, rare disease supplementary concept word, unique identifier, synonyms] 4765

46 (("upper extremity" adj3 questionnaire) or ("upper extremity" adj3 instrument) or ("upper extremity" adj3 scale) or ("upper extremity" adj3 index) or ("upper extremity" adj3 score) or ("upper extremity" adj3 measure) or ("upper extremity" adj3 tool)).mp. [mp=title, abstract, original title, name of substance word, subject heading word, floating sub-heading word, keyword heading word, organism supplementary concept word, protocol supplementary concept word, rare disease supplementary concept word, unique identifier, synonyms] 743

47 (("lower extremity" adj3 questionnaire) or ("lower extremity" adj3 instrument) or ("lower extremity" adj3 scale) or ("lower extremity" adj3 index) or ("lower extremity" adj3 score) or ("lower extremity" adj3 measure) or ("lower extremity" adj3 tool)).mp. [mp=title, abstract, original title, name of substance word, subject heading word, floating sub-heading word, keyword heading word, organism supplementary concept word, protocol supplementary concept word, rare disease supplementary concept word, unique identifier, synonyms] 1190

48 (("public transport*" adj3 questionnaire) or ("public transport*" adj3 instrument) or ("public transport*" adj3 scale) or ("public transport*" adj3 index) or ("public transport*" adj3 score) or ("public transport*" adj3 measure) or ("public transport*" adj3 tool)).mp. [mp=title, abstract, original title, name of substance word, subject heading word, floating sub-heading word, keyword heading word, organism supplementary concept word, protocol supplementary concept word, rare disease supplementary concept word, unique identifier, synonyms] 9

49 35 or 36 or 37 or 38 or 39 or 40 or 41 or 42 or 43 or 44 or 45 or 46 or 47 or 48 180527

50 exp Aged/ 3388630

51 (older* or senior* or geriatri* or elder* or aging or ageing).mp. 1119547

52 50 or 51 3990825

53 exp self report/ 40129

54 exp patient reported outcome measures/ 11269

55 (patient? rating? or patient? report* or subject* report? or subject* rating? or self report* or self evaluation? or self appraisal? or self assess* or self rating? or self ratd).mp. 324540

56 53 or 54 or 55 324597

57 34 and 49 and 52 and 56 6091

58 (addresses or biography or "case reports" or comment or directory or editorial or festschrift or interview or lectures or "legal cases" or legislation or letter or news or "newspaper article" or "patient education handout" or "popular works" or congresses or "consensus development conference" or "consensus development conference, nih" or "practice guideline").pt. 4543100

59 exp animals/ not exp humans/ 4983395

60 57 not 58 not 59 6065

**Embase <1996 to 2022 April 04>**

1 (instrumentation or methods).mp. 3563898

2 ("validation study" or "comparative study").mp. 855486

3 exp Psychometrics/ 94091

4 psychometr*.tw. 60840

5 (clinimetr* or clinometr*).mp. 2043

6 exp "Outcome Assessment, Health Care"/ 678219

7 "outcome assessment".tw. 5948

8 "outcome measure*".mp. 323281

9 exp "Observer Variation"/ 18927

10 "observer variation".tw. 1354

11 exp "Health Status Indicators"/ 37497

12 exp "Reproducibility of Results"/ 231669

13 reproducib*.tw. 192724

14 exp "Discriminant Analysis"/ 21713

15 (reliab* or unreliab* or valid* or "coefficient of variation" or coefficient or homogeneity or homogeneous or "internal consistency").tw. 1947163

16 (cronbach* and (alpha or alphas)).tw. 33039

17 (item and (correlation* or selection* or reduction*)).tw. 35505

18 agreement.mp. 313778

19 precision.mp. 191141

20 imprecision.mp. 9059

21 "precise values".mp. 223

22 test-retest.tw. 33743

23 (test and retest).tw. 35518

24 (reliab* and (test or retest)).tw. 124394

25 stability.tw. 510278

26 (interrater or inter-rater or intrarater or intra-rater).tw. 29682

27 (intertester or inter-tester or intratester or intra-tester).tw. 711

28 (interobserver or inter-observer or intraobserver or intra-observer).tw. 41179

29 (intertechnician or inter-technician or intratechnician or intra-technician).tw. 37

30 (interexaminer or inter-examiner or intraexaminer or intra-examiner).tw. 2004

31 (interassay or inter-assay or intraassay or intra-assay).tw. 10650

32 (interindividual or inter-individual or intraindividual or intra-individual).tw. 43454

33 (interparticipant or inter-participant or intraparticipant or intra-participant).tw. 223

34 kappa.tw. 111530

35 kappas.tw. 1063

36 repeatab*.mp. 51021

37 ((replicab* or repeated) and (measure or measures or findings or result or results or test or tests)).mp. 298110

38 (generaliza* or generalisa*).tw. 55312

39 concordance.tw. 77371

40 (intraclass and correlation*).tw. 35591

41 discriminative.tw. 24772

42 "known group".tw. 1512

43 ("factor analysis" or "factor analyses" or "factor structure" or "factor structures").tw. 64937

44 dimension*.tw. 608901

45 subscale*.tw. 68947

46 (multitrait and scaling and (analysis or analyses)).tw. 150

47 "item discriminant".tw. 129

48 "interscale correlation*".tw. 161

49 (error or errors).tw. 393653

50 "individual variability".tw. 12366

51 "interval variability".tw. 750

52 "rate variability".tw. 28352

53 (variability and (analysis or values)).tw. 146199

54 (uncertainty and (measurement or measuring)).tw. 9920

55 "standard error of measurement".tw. 2711

56 sensitiv*.tw. 1627516

57 responsive*.tw. 253473

58 (limit and detection).tw. 112976

59 "minimal detectable concentration".tw. 63

60 interpretab*.tw. 14669

61 ((minimal or minimally or clinical or clinically) and (important or significant or detectable) and (change or difference)).tw. 448807

62 (small* and (real or detectable) and (change or difference)).tw. 12669

63 "meaningful change".tw. 2182

64 "ceiling effect".tw. 2757

65 "floor effect".tw. 976

66 "item response model".tw. 142

67 IRT.tw. 4725

68 rasch.tw. 5812

69 "differential item functioning".tw. 2355

70 DIF.tw. 5007

71 "computer adaptive testing".tw. 334

72 "item bank".tw. 963

73 "cross-cultural equivalence".tw. 143

74 or/1-73 9413727

75 ((mobil* adj3 questionnaire) or (mobil* adj3 instrument) or (mobil* adj3 scale) or (mobil* adj3 index) or (mobil* adj3 score) or (mobil* adj3 measure) or (mobil* adj3 tool)).mp. or exp mobility limitation/ 20523

76 ((move* adj3 questionnaire) or (move* adj3 instrument) or (move* adj3 scale) or (move* adj3 index) or (move* adj3 score) or (move* adj3 measure) or (move* adj3 tool)).mp. [mp=title, abstract, heading word, drug trade name, original title, device manufacturer, drug manufacturer, device trade name, keyword heading word, floating subheading word, candidate term word] 7340

77 ((abilit* adj3 questionnaire) or (abilit* adj3 instrument) or (abilit* adj3 scale) or (abilit* adj3 index) or (abilit* adj3 score) or (abilit* adj3 measure) or (abilit* adj3 tool)).mp. [mp=title, abstract, heading word, drug trade name, original title, device manufacturer, drug manufacturer, device trade name, keyword heading word, floating subheading word, candidate term word] 14268

78 ((walk* adj3 questionnaire) or (walk* adj3 instrument) or (walk* adj3 scale) or (walk* adj3 index) or (walk* adj3 score) or (walk* adj3 measure) or (walk* adj3 tool)).mp. [mp=title, abstract, heading word, drug trade name, original title, device manufacturer, drug manufacturer, device trade name, keyword heading word, floating subheading word, candidate term word] 5396

79 ((limitation? adj3 questionnaire) or (limitation? adj3 instrument) or (limitation? adj3 scale) or (limitation? adj3 index) or (limitation? adj3 score) or (limitation? adj3 measure) or (limitation? adj3 tool)).mp. [mp=title, abstract, heading word, drug trade name, original title, device manufacturer, drug manufacturer, device trade name, keyword heading word, floating subheading word, candidate term word] 3769

80 ((function* adj3 questionnaire) or (function* adj3 instrument) or (function* adj3 scale) or (function* adj3 index) or (function* adj3 score) or (function* adj3 measure) or (function* adj3 tool)).mp. [mp=title, abstract, heading word, drug trade name, original title, device manufacturer, drug manufacturer, device trade name, keyword heading word, floating subheading word, candidate term word] 104780

81 ((transfer* adj3 questionnaire) or (transfer* adj3 instrument) or (transfer* adj3 scale) or (transfer* adj3 index) or (transfer* adj3 score) or (transfer* adj3 measure) or (transfer* adj3 tool)).mp. [mp=title, abstract, heading word, drug trade name, original title, device manufacturer, drug manufacturer, device trade name, keyword heading word, floating subheading word, candidate term word] 3511

82 ((ambulat* adj3 questionnaire) or (ambulat* adj3 instrument) or (ambulat* adj3 scale) or (ambulat* adj3 index) or (ambulat* adj3 score) or (ambulat* adj3 measure) or (ambulat* adj3 tool)).mp. [mp=title, abstract, heading word, drug trade name, original title, device manufacturer, drug manufacturer, device trade name, keyword heading word, floating subheading word, candidate term word] 2366

83 ((activit* adj3 questionnaire) or (activit* adj3 instrument) or (activit* adj3 scale) or (activit* adj3 index) or (activit* adj3 score) or (activit* adj3 measure) or (activit* adj3 tool)).mp. [mp=title, abstract, heading word, drug trade name, original title, device manufacturer, drug manufacturer, device trade name, keyword heading word, floating subheading word, candidate term word] 96200

84 ((disabilit* adj3 questionnaire) or (disabilit* adj3 instrument) or (disabilit* adj3 scale) or (disabilit* adj3 index) or (disabilit* adj3 score) or (disabilit* adj3 measure) or (disabilit* adj3 tool)).mp. [mp=title, abstract, heading word, drug trade name, original title, device manufacturer, drug manufacturer, device trade name, keyword heading word, floating subheading word, candidate term word] 56725

85 ((exercise adj3 questionnaire) or (exercise adj3 instrument) or (exercise adj3 scale) or (exercise adj3 index) or (exercise adj3 score) or (exercise adj3 measure) or (exercise adj3 tool)).mp. [mp=title, abstract, heading word, drug trade name, original title, device manufacturer, drug manufacturer, device trade name, keyword heading word, floating subheading word, candidate term word] 6576

86 (("upper extremity" adj3 questionnaire) or ("upper extremity" adj3 instrument) or ("upper extremity" adj3 scale) or ("upper extremity" adj3 index) or ("upper extremity" adj3 score) or ("upper extremity" adj3 measure) or ("upper extremity" adj3 tool)).mp. [mp=title, abstract, heading word, drug trade name, original title, device manufacturer, drug manufacturer, device trade name, keyword heading word, floating subheading word, candidate term word] 1104

87 (("lower extremity" adj3 questionnaire) or ("lower extremity" adj3 instrument) or ("lower extremity" adj3 scale) or ("lower extremity" adj3 index) or ("lower extremity" adj3 score) or ("lower extremity" adj3 measure) or ("lower extremity" adj3 tool)).mp. [mp=title, abstract, heading word, drug trade name, original title, device manufacturer, drug manufacturer, device trade name, keyword heading word, floating subheading word, candidate term word] 1745

88 (("public transport*" adj3 questionnaire) or ("public transport*" adj3 instrument) or ("public transport*" adj3 scale) or ("public transport*" adj3 index) or ("public transport*" adj3 score) or ("public transport*" adj3 measure) or ("public transport*" adj3 tool)).mp. [mp=title, abstract, heading word, drug trade name, original title, device manufacturer, drug manufacturer, device trade name, keyword heading word, floating subheading word, candidate term word] 11

89 75 or 76 or 77 or 78 or 79 or 80 or 81 or 82 or 83 or 84 or 85 or 86 or 87 or 88 298971

90 exp Aged/ 2855618

91 (older* or senior* or geriatri* or elder* or aging or ageing).mp. 1608602

92 90 or 91 3708594

93 exp self report/ 135415

94 exp patient reported outcome measures/ 39348

95 (patient? rating? or patient? report* or subject* report? or subject* rating? or self report* or self evaluation? or self appraisal? or self assess* or self rating? or self ratd).mp. 465354

96 93 or 94 or 95 465990

97 (addresses or biography or "case reports" or comment or directory or editorial or festschrift or interview or lectures or "legal cases" or legislation or letter or news or "newspaper article" or "patient education handout" or "popular works" or congresses or "consensus development conference" or "consensus development conference, nih" or "practice guideline").pt. 1568787

98 exp animals/ not exp humans/ 3351826

99 74 and 89 and 92 and 96 7167

100 99 not 97 not 98 7157

**CINAHL**

| **#** | **Query** | **Results** |
| --- | --- | --- |
| S9 | S8 NOT S7 | 3,492 |
| S8 | S1 AND S2 AND S3 AND S4 | 3,498 |
| S7 | S5 OR S6 | 841,560 |
| S6 | (MH animals+) NOT (MH humans+) | 99,644 |
| S5 | (PT addresses OR PT biography OR PT "case reports" OR PT comment OR PT directory OR PT editorial OR PT festschrift OR PT interview OR PT lectures OR PT "legal cases" OR PT legislation OR PT letter OR PT news OR PT "newspaper article" OR PT "patient education handout" OR PT "popular works" OR PT congresses OR PT "consensus development conference" OR PT "consensus development conference, nih" OR PT "practice guideline") | 753,179 |
| S4 | (MH Aged+) OR (old* OR senior* OR geriatri* OR elder* OR ageing OR aging) | 1,183,050 |
| S3 | ((mobil* N3 questionnaire) OR (mobil* N3 instrument) OR (mobil* N3 scale) OR (mobil* N3 index) OR (mobil* N3 score) OR (mobil* N3 measure) OR (mobil* N3 tool)) OR ((move* N3 questionnaire) OR (move* N3 instrument) OR (move* N3 scale) OR (move* N3 index) OR (move* N3 score) OR (move* N3 measure) OR (move* N3 tool)) OR ((abilit* N3 questionnaire) OR (abilit* N3 instrument) OR (abilit* N3 scale) OR (abilit* N3 index) OR (abilit* N3 score) OR (abilit* N3 measure) OR (abilit* N3 tool)) OR ((walk* N3 questionnaire) OR (walk* N3 instrument) OR (walk* N3 scale) OR (walk* N3 index) OR (walk* N3 score) OR (walk* N3 measure) OR (walk* N3 tool)) OR ((limitation# N3 questionnaire) OR (limitation# N3 instrument) OR (limitation# N3 scale) OR (limitation# N3 index) OR (limitation# N3 score) OR (limitation# N3 measure) OR (limitation# N3 tool)) OR ((function* N3 questionnaire) OR (function* N3 instrument) OR (function* N3 scale) OR (function* N3 index) OR (function* N3 score) OR (function* N3 measure) OR (function* N3 tool)) OR ((transfer* N3 questionnaire) OR (transfer* N3 instrument) OR (transfer* N3 scale) OR (transfer* N3 index) OR (transfer* N3 score) OR (transfer* N3 measure) OR (transfer* N3 tool)) ((ambulat* N3 questionnaire) OR (ambulat* N3 instrument) OR (ambulat* N3 scale) OR (ambulat* N3 index) OR (ambulat* N3 score) OR (ambulat* N3 measure) OR (ambulat* N3 tool)) OR ((activit* N3 questionnaire) OR (activit* N3 instrument) OR (activit* N3 scale) OR (activit* N3 index) OR (activit* N3 score) OR (activit* N3 measure) OR (activit* N3 tool)) OR ((disabilit* N3 questionnaire) OR (disabilit* N3 instrument) OR (disabilit* N3 scale) OR (disabilit* N3 index) OR (disabilit* N3 score) OR (disabilit* N3 measure) OR (disabilit* N3 tool)) OR ((exercise N3 questionnaire) OR (exercise N3 instrument) OR (exercise N3 scale) OR (exercise N3 index) OR (exercise N3 score) OR (exercise N3 measure) OR (exercise N3 tool)) OR (("upper extremity" N3 questionnaire) OR ("upper extremity" N3 instrument) OR ("upper extremity" N3 scale) OR ("upper extremity" N3 index) OR ("upper extremity" N3 score) OR ("upper extremity" N3 measure) OR ("upper extremity" N3 tool)) OR (("lower extremity" N3 questionnaire) OR ("lower extremity" N3 instrument) OR ("lower extremity" N3 scale) OR ("lower extremity" N3 index) OR ("lower extremity" N3 score) OR ("lower extremity" N3 measure) OR ("lower extremity" N3 tool)) OR (("public transport*" N3 questionnaire) OR ("public transport*" N3 instrument) OR ("public transport*" N3 scale) OR ("public transport*" N3 index) OR ("public transport*" N3 score) OR ("public transport*" N3 measure) OR ("public transport*" N3 tool)) | 106,993 |
| S2 | ((MH "self report"+) ) OR ((MH "patient reported outcome measures"+) ) OR (("patient# rating#" OR "subject* report#" OR "subject* rating#" OR "self report*" OR "self evaluation#" OR "self appraisal#" OR "self assess*" OR "self rating#" OR "self rated")) OR (("patient# report*")) | 191,670 |
| S1 | (MH "Psychometrics") or ( TI psychometr* or AB psychometr* ) or ( TI clinimetr* or AB clinimetr* ) or ( TI clinometr* OR AB clinometr* ) or (MH "Outcome Assessment") or ( TI outcome assessment or AB outcome assessment ) or ( TI outcome measure* or AB outcome measure* ) or (MH "Health Status Indicators") or (MH "Reproducibility of Results") or (MH"Discriminant Analysis") or ( ( TI reproducib* or AB reproducib* ) or ( TI reliab* or AB reliab* ) or ( TI unreliab* or AB unreliab* ) ) or ( ( TI valid* or AB valid* ) or ( TI coefficient or AB coefficient ) or ( TI homogeneity or AB homogeneity ) ) or ( TI homogeneous or AB homogeneous ) or ( TI "coefficient of variation" or AB "coefficient of variation") or ( TI "internal consistency" or AB "internal consistency") or (MH "Internal Consistency+") or (MH"Reliability+") or (MH "Measurement Error+") or (MH "Content Validity+") or "hypothesis testing" or "structural validity" or "cross-cultural validity" or (MH "Criterion-Related Validity+") or "responsiveness" or "interpretability" or ( TI reliab* or AB reliab* ) and ( (TI test or AB test) OR (TI retest or AB retest) ) or ( TI stability or AB stability ) or ( TI interrater or AB interrater ) or ( TI inter-rater or AB inter-rater ) or ( TI intrarater or AB intrarater ) or ( TI intra-rater or AB intrarater) or ( TI intertester or AB intertester) or (TI inter-tester or AB inter-tester) or ( TI intratester or AB intratester) or ( TI intra-tester or AB intra-tester) or ( TI interobserver or AB interobserver) or (TI inter-observer or AB inter-observer ) or ( TI intraobserver or AB intraobserver) or ( TI intra-observer or AB intra-observer) or ( TI intertechnician or AB intertechnician) or (TI inter-technician or AB inter-technician) or ( TI intratechnician or AB intratechnician ) or ( TI intra-technician or AB intra-technician ) or ( TI interexaminer or AB interexaminer ) or (TI inter-examiner or AB inter-examiner) or (TI intraexaminer or AB intraexaminer ) OR (TI intra-examiner or AB intra-examiner ) or (TI intra-examiner or AB intraexaminer) or (TI interassay or AB interassay ) or ( TI inter-assay or AB inter-assay ) or ( TI intraassay or AB intraassay) or ( TI intra-assay or AB intra-assay) or (TI interindividual or AB interindividual) or (TI inter-individual or AB inter-individual) OR (TI intraindividual or AB intraindividual) or (TI intra-individual or AB intra-individual) or (TI interparticipant or AB interparticipant) or (TI inter-participant or AB inter-participant ) or (TI intraparticipant or AB intraparticipant) or (TI intra-participant or AB intra-participant ) or (TI kappa or AB kappa) or (TI kappa's or AB kappa's ) or (TI kappas or AB kappas) or (TI repeatab* or AB repeatab*) or ( TI responsive* or AB responsive*) or ( TI interpretab* or AB interpretab*) | 675,901 |

**APA PsycInfo <1987 to March Week 4 2022>**

1 (instrumentation or methods).hw. 78979

2 ("Validation Studies" or "Comparative Study").ti,ab. 10832

3 exp Psychometrics/ 188211

4 psychometr*.ti,ab. 57260

5 (clinimetr* or clinometr*).ti,ab. 304

6 "outcome assessment".ti,ab. 1000

7 "outcome measure*".ti,ab. 40336

8 "observer variation".ti,ab. 13

9 "Health Status Indicators".mp. 3850

10 exp Test Reliability/ or exp Test Validity/ 95250

11 reproducib*.ti,ab. 6023

12 exp Discrimination/ 38060

13 (reliab* or unreliab* or valid* or coefficient or homogeneity or homogeneous or "internal consistency").ti,ab. 330593

14 (cronbach* and (alpha or alphas)).ti,ab. 14437

15 (item and (correlation* or selection* or reduction*)).ti,ab. 17977

16 (agreement or precision or imprecision or "precise values" or test-retest).ti,ab. 66046

17 (test and retest).ti,ab. 18206

18 (reliab* and (test or retest)).ti,ab. 37737

19 (stability or interrater or inter-rater or intrarater or intra-rater or intertester or inter-tester or intratester or intra-tester or interobserver or inter-observer or intraobserver or intraobserver or intertechnician or inter-technician or intratechnician or intra-technician or interexaminer or inter-examiner or intraexaminer or intra-examiner or interassay or interassay or intraassay or intra-assay or interindividual or inter-individual or intraindividual or intra-individual or interparticipant or inter-participant or intraparticipant or intra-participant or kappa or kappa's or kappas or epeatab*).ti,ab. 66645

20 ((replicab* or repeated) and (measure or measures or findings or result or results or test or tests)).ti,ab. 50803

21 (generaliza* or generalisa* or concordance).ti,ab. 40360

22 (intraclass and correlation*).ti,ab. 4856

23 (discriminative or "known group" or "factor analysis" or "factor analyses" or dimension* or subscale*).ti,ab. 246181

24 (multitrait and scaling and (analysis or analyses)).ti,ab. 57

25 ("item discriminant" or "interscale correlation*" or error or errors or "individual variability").ti,ab. 103348

26 (variability and (analysis or values)).ti,ab. 13934

27 (uncertainty and (measurement or measuring)).ti,ab. 1076

28 ("standard error of measurement" or sensitiv* or responsive*).ti,ab. 191586

29 ((minimal or minimally or clinical or clinically) and (important or significant or detectable) and (change or difference)).ti,ab. 29142

30 (small* and (real or detectable) and (change or difference)).ti,ab. 1035

31 ("meaningful change" or "ceiling effect" or "floor effect" or "Item response model" or IRT or Rasch or "Differential item functioning" or DIF or "computer adaptive testing" or "item bank" or "cross-cultural equivalence").ti,ab. 12237

32 or/1-31 1093678

33 ((mobil* adj3 questionnaire) or (mobil* adj3 instrument) or (mobil* adj3 scale) or (mobil* adj3 index) or (mobil* adj3 score) or (mobil* adj3 measure) or (mobil* adj3 tool)).mp. or exp mobility limitation/ 1653

34 ((move* adj3 questionnaire) or (move* adj3 instrument) or (move* adj3 scale) or (move* adj3 index) or (move* adj3 score) or (move* adj3 measure) or (move* adj3 tool)).mp. [mp=title, abstract, heading word, table of contents, key concepts, original title, tests & measures, mesh word] 3971

35 ((abilit* adj3 questionnaire) or (abilit* adj3 instrument) or (abilit* adj3 scale) or (abilit* adj3 index) or (abilit* adj3 score) or (abilit* adj3 measure) or (abilit* adj3 tool)).mp. [mp=title, abstract, heading word, table of contents, key concepts, original title, tests & measures, mesh word] 7295

36 ((walk* adj3 questionnaire) or (walk* adj3 instrument) or (walk* adj3 scale) or (walk* adj3 index) or (walk* adj3 score) or (walk* adj3 measure) or (walk* adj3 tool)).mp. [mp=title, abstract, heading word, table of contents, key concepts, original title, tests & measures, mesh word] 1170

37 ((limitation? adj3 questionnaire) or (limitation? adj3 instrument) or (limitation? adj3 scale) or (limitation? adj3 index) or (limitation? adj3 score) or (limitation? adj3 measure) or (limitation? adj3 tool)).mp. [mp=title, abstract, heading word, table of contents, key concepts, original title, tests & measures, mesh word] 1310

38 ((function* adj3 questionnaire) or (function* adj3 instrument) or (function* adj3 scale) or (function* adj3 index) or (function* adj3 score) or (function* adj3 measure) or (function* adj3 tool)).mp. [mp=title, abstract, heading word, table of contents, key concepts, original title, tests & measures, mesh word] 32785

39 ((transfer* adj3 questionnaire) or (transfer* adj3 instrument) or (transfer* adj3 scale) or (transfer* adj3 index) or (transfer* adj3 score) or (transfer* adj3 measure) or (transfer* adj3 tool)).mp. [mp=title, abstract, heading word, table of contents, key concepts, original title, tests & measures, mesh word] 601

40 ((ambulat* adj3 questionnaire) or (ambulat* adj3 instrument) or (ambulat* adj3 scale) or (ambulat* adj3 index) or (ambulat* adj3 score) or (ambulat* adj3 measure) or (ambulat* adj3 tool)).mp. [mp=title, abstract, heading word, table of contents, key concepts, original title, tests & measures, mesh word] 276

41 ((activit* adj3 questionnaire) or (activit* adj3 instrument) or (activit* adj3 scale) or (activit* adj3 index) or (activit* adj3 score) or (activit* adj3 measure) or (activit* adj3 tool)).mp. [mp=title, abstract, heading word, table of contents, key concepts, original title, tests & measures, mesh word] 18800

42 ((disabilit* adj3 questionnaire) or (disabilit* adj3 instrument) or (disabilit* adj3 scale) or (disabilit* adj3 index) or (disabilit* adj3 score) or (disabilit* adj3 measure) or (disabilit* adj3 tool)).mp. [mp=title, abstract, heading word, table of contents, key concepts, original title, tests & measures, mesh word] 12461

43 ((exercise adj3 questionnaire) or (exercise adj3 instrument) or (exercise adj3 scale) or (exercise adj3 index) or (exercise adj3 score) or (exercise adj3 measure) or (exercise adj3 tool)).mp. [mp=title, abstract, heading word, table of contents, key concepts, original title, tests & measures, mesh word] 3791

44 (("upper extremity" adj3 questionnaire) or ("upper extremity" adj3 instrument) or ("upper extremity" adj3 scale) or ("upper extremity" adj3 index) or ("upper extremity" adj3 score) or ("upper extremity" adj3 measure) or ("upper extremity" adj3 tool)).mp. [mp=title, abstract, heading word, table of contents, key concepts, original title, tests & measures, mesh word] 176

45 (("lower extremity" adj3 questionnaire) or ("lower extremity" adj3 instrument) or ("lower extremity" adj3 scale) or ("lower extremity" adj3 index) or ("lower extremity" adj3 score) or ("lower extremity" adj3 measure) or ("lower extremity" adj3 tool)).mp. [mp=title, abstract, heading word, table of contents, key concepts, original title, tests & measures, mesh word] 154

46 (("public transport*" adj3 questionnaire) or ("public transport*" adj3 instrument) or ("public transport*" adj3 scale) or ("public transport*" adj3 index) or ("public transport*" adj3 score) or ("public transport*" adj3 measure) or ("public transport*" adj3 tool)).mp. [mp=title, abstract, heading word, table of contents, key concepts, original title, tests & measures, mesh word] 14

47 33 or 34 or 35 or 36 or 37 or 38 or 39 or 40 or 41 or 42 or 43 or 44 or 45 or 46 77519

48 exp Aged/ 2176

49 (older* or senior* or geriatri* or elder* or aging or ageing).mp. 282968

50 48 or 49 283134

51 exp self report/ 19953

52 exp patient reported outcome measures/ 504

53 (patient? rating? or patient? report* or subject* report? or subject* rating? or self report* or self evaluation? or self appraisal? or self assess* or self rating? or self ratd).mp. 196548

54 51 or 52 or 53 196548

55 32 and 47 and 50 and 54 742

**AgeLine**

| **#** | **Query** | **Results** |
| --- | --- | --- |
| S5 | ((MH Aged+) OR (old* OR senior* OR geriatri* OR elder* OR ageing OR aging)) AND (S1 AND S2 AND S3 AND S4) | 326 |
| S4 | (MH Aged+) OR (old* OR senior* OR geriatri* OR elder* OR ageing OR aging) | 169,915 |
| S3 | ((mobil* N3 questionnaire) OR (mobil* N3 instrument) OR (mobil* N3 scale) OR (mobil* N3 index) OR (mobil* N3 score) OR (mobil* N3 measure) OR (mobil* N3 tool)) OR ((move* N3 questionnaire) OR (move* N3 instrument) OR (move* N3 scale) OR (move* N3 index) OR (move* N3 score) OR (move* N3 measure) OR (move* N3 tool)) OR ((abilit* N3 questionnaire) OR (abilit* N3 instrument) OR (abilit* N3 scale) OR (abilit* N3 index) OR (abilit* N3 score) OR (abilit* N3 measure) OR (abilit* N3 tool)) OR ((walk* N3 questionnaire) OR (walk* N3 instrument) OR (walk* N3 scale) OR (walk* N3 index) OR (walk* N3 score) OR (walk* N3 measure) OR (walk* N3 tool)) OR ((limitation# N3 questionnaire) OR (limitation# N3 instrument) OR (limitation# N3 scale) OR (limitation# N3 index) OR (limitation# N3 score) OR (limitation# N3 measure) OR (limitation# N3 tool)) OR ((function* N3 questionnaire) OR (function* N3 instrument) OR (function* N3 scale) OR (function* N3 index) OR (function* N3 score) OR (function* N3 measure) OR (function* N3 tool)) OR ((transfer* N3 questionnaire) OR (transfer* N3 instrument) OR (transfer* N3 scale) OR (transfer* N3 index) OR (transfer* N3 score) OR (transfer* N3 measure) OR (transfer* N3 tool)) ((ambulat* N3 questionnaire) OR (ambulat* N3 instrument) OR (ambulat* N3 scale) OR (ambulat* N3 index) OR (ambulat* N3 score) OR (ambulat* N3 measure) OR (ambulat* N3 tool)) OR ((activit* N3 questionnaire) OR (activit* N3 instrument) OR (activit* N3 scale) OR (activit* N3 index) OR (activit* N3 score) OR (activit* N3 measure) OR (activit* N3 tool)) OR ((disabilit* N3 questionnaire) OR (disabilit* N3 instrument) OR (disabilit* N3 scale) OR (disabilit* N3 index) OR (disabilit* N3 score) OR (disabilit* N3 measure) OR (disabilit* N3 tool)) OR ((exercise N3 questionnaire) OR (exercise N3 instrument) OR (exercise N3 scale) OR (exercise N3 index) OR (exercise N3 score) OR (exercise N3 measure) OR (exercise N3 tool)) OR (("upper extremity" N3 questionnaire) OR ("upper extremity" N3 instrument) OR ("upper extremity" N3 scale) OR ("upper extremity" N3 index) OR ("upper extremity" N3 score) OR ("upper extremity" N3 measure) OR ("upper extremity" N3 tool)) OR (("lower extremity" N3 questionnaire) OR ("lower extremity" N3 instrument) OR ("lower extremity" N3 scale) OR ("lower extremity" N3 index) OR ("lower extremity" N3 score) OR ("lower extremity" N3 measure) OR ("lower extremity" N3 tool)) OR (("public transport*" N3 questionnaire) OR ("public transport*" N3 instrument) OR ("public transport*" N3 scale) OR ("public transport*" N3 index) OR ("public transport*" N3 score) OR ("public transport*" N3 measure) OR ("public transport*" N3 tool)) | 7,423 |
| S2 | ((MH Questionnaires+) ) OR ((MH "self report"+) ) OR ((MH "patient reported outcome measures"+) ) OR (("patient# rating#" OR "subject* report#" OR "subject* rating#" OR "self report*" OR "self evaluation#" OR "self appraisal#" OR "self assess*" OR "self rating#" OR "self rated")) OR (("patient# report*")) | 8,656 |
| S1 | (MH "Psychometrics") or ( TI psychometr* or AB psychometr* ) or ( TI clinimetr* or AB clinimetr* ) or ( TI clinometr* OR AB clinometr* ) or (MH "Outcome Assessment") or ( TI outcome assessment or AB outcome assessment ) or ( TI outcome measure* or AB outcome measure* ) or (MH "Health Status Indicators") or (MH "Reproducibility of Results") or (MH"Discriminant Analysis") or ( ( TI reproducib* or AB reproducib* ) or ( TI reliab* or AB reliab* ) or ( TI unreliab* or AB unreliab* ) ) or ( ( TI valid* or AB valid* ) or ( TI coefficient or AB coefficient ) or ( TI homogeneity or AB homogeneity ) ) or ( TI homogeneous or AB homogeneous ) or ( TI "coefficient of variation" or AB "coefficient of variation") or ( TI "internal consistency" or AB "internal consistency") or (MH "Internal Consistency+") or (MH"Reliability+") or (MH "Measurement Error+") or (MH "Content Validity+") or "hypothesis testing" or "structural validity" or "cross-cultural validity" or (MH "Criterion-Related Validity+") or "responsiveness" or "interpretability" or ( TI reliab* or AB reliab* ) and ( (TI test or AB test) OR (TI retest or AB retest) ) or ( TI stability or AB stability ) or ( TI interrater or AB interrater ) or ( TI inter-rater or AB inter-rater ) or ( TI intrarater or AB intrarater ) or ( TI intra-rater or AB intrarater) or ( TI intertester or AB intertester) or (TI inter-tester or AB inter-tester) or ( TI intratester or AB intratester) or ( TI intra-tester or AB intra-tester) or ( TI interobserver or AB interobserver) or (TI inter-observer or AB inter-observer ) or ( TI intraobserver or AB intraobserver) or ( TI intra-observer or AB intra-observer) or ( TI intertechnician or AB intertechnician) or (TI inter-technician or AB inter-technician) or ( TI intratechnician or AB intratechnician ) or ( TI intra-technician or AB intra-technician ) or ( TI interexaminer or AB interexaminer ) or (TI inter-examiner or AB inter-examiner) or (TI intraexaminer or AB intraexaminer ) OR (TI intra-examiner or AB intra-examiner ) or (TI intra-examiner or AB intraexaminer) or (TI interassay or AB interassay ) or ( TI inter-assay or AB inter-assay ) or ( TI intraassay or AB intraassay) or ( TI intra-assay or AB intra-assay) or (TI interindividual or AB interindividual) or (TI inter-individual or AB inter-individual) OR (TI intraindividual or AB intraindividual) or (TI intra-individual or AB intra-individual) or (TI interparticipant or AB interparticipant) or (TI inter-participant or AB inter-participant ) or (TI intraparticipant or AB intraparticipant) or (TI intra-participant or AB intra-participant ) or (TI kappa or AB kappa) or (TI kappa's or AB kappa's ) or (TI kappas or AB kappas) or (TI repeatab* or AB repeatab*) or ( TI responsive* or AB responsive*) or ( TI interpretab* or AB interpretab*) | 15,428 |

## Appendix B. Characteristics of Self-report Perceived Mobility Measures included in the Review

| **Measure** | **Framework** | **Number of items** | **Total score** | **Total Score interpretation** | **Subscales or extended measures** |
| --- | --- | --- | --- | --- | --- |
| Late Life Function and Disability Index: function component (LLFD-FC) | Developed based on Nagi’s disablement model for older adults in English (Hebrew version and Swedish versions were included) | 32 (upper extremity: 7 items; basic lower extremity: 14 items; advanced lower extremity: 11 items) | 0-100 | Higher scores indicate better function levels | Overall score  Upper extremity  Basic lower extremity  Advanced lower extremity |
| Lower extremity functional scale (LEFS) | Unclear framework; developed for people with musculoskeletal conditions in English | 20 | 0-80 | Higher scores indicate better function levels | NA |
| Perceived Driving Abilities (PDA) | Unclear framework: developed for older adults to assess a person’s perceived driving abilities in English (Canada) | 15 | 0-45 | Higher scores indicate better ability | Current perceived driving ability  Changes in abilities over the past 10 years |
| **Mobility Assessment Tool (MAT)**  Self-report tool (video-based) | Unclear framework: developed in English (Portuguese and Spanish version were included in the review) | 10 or 12 items for MAT-short form; 2 for MAT-Walking | MAT-SF: 30-80  MAT-walking: walking speed and walking distance | Higher scores indicate better ability levels | MAT-short form (MAT-SF)  MAT-Walking |
| **Questionnaire Rising and Sitting down (QR&S)** | Unclear framework; developed in Dutch | 39 items | 0-100 | Higher scores indicate better function levels | NA |
| Patient-reported outcomes measurement information system (PROMIS) physical function | Developed based on International Classification System of Functioning, Disability and Health (ICF) framework | Items bank | T-score | Higher scores indicate better function levels | NA |
| Modified Rosow-Breslau scale | Unclear framework | 4 | 0-4 | Higher scores indicate better function levels | NA |
| Self-reported measures of functional status – mobility dimension, adapted from the WHO questionnaire | Unclear framework | 4 | Categorical | NA | NA |
| Nagi items (9), Rosow-Breslau items (2), SPPARCS items (2) | Unclear framework | 13 | Categorical | NA | NA |
| Self-reported walking index (RW-index) | Unclear framework | 5 | 4-11 | Higher scores indicate better function levels | NA |
| Preclinical and manifest mobility limitation | Development of mobility limitation | 3 | Categorical | NA | NA |
| Pepper Assessment Tool for Disability (PAT-D): mobility domain | The International Classification System of Functioning, Disability and Health (ICF) framework | 6 | 6-30 | Higher scores indicate worse function levels | NA |
| Self-report physical capacity measures in National Health and Aging Trends Study (NHATS) | Unclear framework | 6 | Categorical | NA | NA |
| Self-Reported Measure of Function (mobility domain) | Unclear framework | 3 | Categorical | NA | NA |
| Self-reported measures of walking, running, and lifting abilities | Activity Space Model (ASM) of disability | Walking: 5; Running: 5; Lifting: 6 | 0-100 | Higher scores indicate better function levels | NA |
| Self-report major mobility disability | Unclear framework | 3 | Categorical | NA | NA |
| Lower Extremity Computerized Adaptive Test (LE-CAT). | Unclear framework | Items bank | NA | Higher scores indicate better function levels | NA |

NA: not applicable.

## Appendix C. Criteria for good measurement properties and related hypothesis

| Psychometric Property | Rating | Criteria |
| --- | --- | --- |
| Internal Consistency | + | Cronbach’s alpha(s) ≥ 0.70 for each unidimensional scale or subscale |
|  | ? | Cronbach’s alpha not reported |
|  | - | Cronbach’s alpha(s) < 0.70 for each unidimensional scale or subscale |
| Reliability | + | ICC, weighted Kappa, or correlations ≥0.70 |
|  | ? | ICC, weighted Kappa, or correlations not reported |
|  | - | ICC, weighted Kappa, or correlation <0.70 |
| Measurement Error | + | SDC or LoA <MIC |
|  | ? | MIC not defined |
|  | - | SDC or LoA >MIC |
| Hypothesis for Construct Validity | + | Results in accordance with hypothesis |
|  | ? | No statistics reported* |
|  | - | Results not in accordance with the hypothesis |
| Structure Validity | + | Evidence support |
|  | ? | No statistics reported |
|  | - | Correlation with gold standard < 0.70 OR AUC < 0.70 |
| Responsiveness | + | The result is in accordance with the hypothesis OR AUC ≥ 0.70* |
|  | ? | No statistics reported* |
|  | - | Results not in accordance with the hypothesis OR AUC < 0.70 |

The criteria are based on COSMIN criteria.

AUC = area under the curve, ICC = intraclass correlation coefficient, IRT = item response theory, LoA = limits of agreement, MIC = minimal important change, SDC = smallest detectable change

*This criteria has been modified from the COSMIN criteria for good measurement properties as the review team defined all hypotheses

**Hypothesis for measures with good psychometric properties**

**Convergent validity**

We hypothesized that a correlation coefficient of at least 0.5 would be observed between self-reported mobility measures and conceptually related constructs such as physical function or disability measures, performance-based mobility measures, and physical activity measures. A correlation coefficient of at least 0.3 would be observed between self-reported mobility measures and conceptually relevant constructs such as fall-efficacy scale, depression, or balance measures.

For measures of perceived driving ability, we hypothesized that current ability would have a correlation coefficient of at least 0.5 with actual driving ability, driver comfort level, driving restrictions and driving behaviours, and hypothesized that the changes of perceived driving would have a correlation coefficient of at least 0.3.

**Known-group validity**

We hypothesized that older adults with more functional limitations at pre-treatment would have lower perceived mobility abilities than adults with higher function levels or after-treatment and hypothesized that the group differences would be at least statistically significant (*p* < 0.05).

**Predictive validity**

We hypothesized that self-reported mobility measures could statistically predict adverse outcomes with an area under the curve (AUC) of at least 0.70.

**Responsiveness**

We hypothesized that the changes over time would be greater or equal to the minimal important difference, and hypothesized that the effect size of mobility measure change scores would be small and significantly correlated with the external anchor, at least with r = 0.3.

## Appendix D. Characteristics of the 36 eligible studies

| **Study** | **Country** | **Study design** | **Follow up (mean or median months)** | **Setting** | **N** | **Age Mean or median** | **Gender (female)** | **Mobility measures** | **Reported psychometric properties** |
| --- | --- | --- | --- | --- | --- | --- | --- | --- | --- |
| Haley 2002 | USA | Cross-sectional and test-retest | Test-retest: within 1-3 weeks, 12 days (SD 8.3) | Community | 150 (15 for test-retest reliability) | 76 | 77.30% | LLFDI-FC; upper extremity; basic lower extremity; advanced lower extremity | Internal consistency; Reliability; Convergent validity, Known-groups validity |
| Sayers 2004 | USA | Cross-sectional | NA | Community | 101 | 81 | 63.00% | LLFDI-FC; upper extremity; basic lower extremity; advanced lower extremity | Convergent validity |
| Melzer 2007 | Israel | Cross-sectional and test-retest reliability | 10-14 days | Community | 55 | 80 | 76.36% | LLFDI-FC; upper extremity; basic lower extremity; advanced lower extremity | Reliability; Convergent validity, Known-groups validity |
| Lapier 2012 | USA | Cross-sectional and test-retest reliability | 1-5days | Outpatient | 29 | 69 | 28.00% | LLFDI-FC (self reported and interview form) | Convergent validity; Floor/ceiling effects; Change score |
| Feuering 2014 | Isreal | Cross-sectional | NA | Mixed (50% Community) | 50 | 80 | 64.00% | LLFDI-FC upper extremity subscale and basic lower extremity subscale | Reliability; Convergent validity |
| Roaldsen 2014 | Sweden | Test-retest reliability | 2 weeks for reliability | Community (Control group in a clinical trial) | 62 | 76 | 87.00% | LLFDI-FC; upper extremity; basic lower extremity; advanced lower extremity | Internal consistency; Reliability; Convergent validity |
| Beauchamp 2015 | USA | Prospective cohort | 2 years | Community (primary care patients) | 430 | 76.6 | 68.00% | LLFDI-FC; upper extremity; basic lower extremity; advanced lower extremity | MDC; SEM; Predictive validity; Responsiveness |
| Beauchamp 2019 | USA | Prospective cohort | 1 year (between 2 and 3 years) | Community (primary care patients) | 320 | 76.2 | 68.50% | LLFDI-FC; upper extremity; basic lower extremity; advanced lower extremity | MID; MDC; SEM; Responsiveness |
| O'Hoski 2020 | USA | Prospective cohort | 2 years | Community (primary care patients) | 391 | 76.5 | 66.70% | LLFDI-FC; basic lower extremity; advanced lower extremity | Predictive validity |
| Verheijde 2013 | USA | Prospective cohort | 8 weeks and 4 weeks | Mixed (outpatients:77% and inpatients) | 43 | 70 | 23.00% | Lower Extremity Functional Scale (LEFS) | Reliability; Convergent validity; Change scores |
| Paravlic 2020 | Slovenia | Cross-sectional and test-retest reliability | about 1 week for reliability (6.8±0.7 days) | Mixed (separately reported community and inpatients) | Total: 123; eligible participants: 45 | 65.87 | 68.89% | LEFS | Internal consistency; Reliability; MDC; SEM |
| Stratford 2009 | Canada | A known-group construct validation design | NA | Community (part of cohort study) | 73 | 61.9 | 38.36% | LEFS | Known-groups validity |
| Pua 2009 | Australia | Cross-sectional and test-retest | about 1 week for reliability | Community (part of a cross-sectional study) | 100 (n=43 for test-retest) | 62 | 60.00% | LEFS | Reliability; MDC; SEM; Convergent validity |
| Chen 2021 | Canada | Cross-sectional | NA | Community (part of Candrive study) | 108 | 81 | 32.40% | Perceived Driving Ability Questionnaire (PDA) | Accuracy of self perceived and objective measures |
| MacDonald 2008 | Canada | Retrospective cohort | 10 years for change | Community (including seniors' centers) | Total: 71; with functional assessment: n=42 | 78.2 | 56.30% | PDA current and change scores | Internal consistency; Convergent validity |
| Blanchard 2010 | Canada | Cross-sectional and test-retest (about one week apart) | about 1 week | Community (including seniors' centers) | psychometric purpose： 39 | 73.6 | 53.80% | PDA current and change scores | Internal consistency; Reliability; Convergent validity |
| Rejeski 2010 | USA | Longitudinal study | two weeks for test-retest reliability | Community (including continuing care retirement communities) | 234 (reliability: n=30) | 82 | 70.90% | Mobility Assessment Tool-short form (MAT-sf) | Reliability; Convergent validity, Known-groups validity |
| Rejeski 2013 | USA | Cross-sectional | NA | Community | 1,343 | 79 | 70.20% | mobility assessment tool—short form (MAT-sf) | Covergent validity; Floor/ceiling effects; Known-groups |
| Guerra 2014 | Brazil and Colombia | Cross-sectional | NA (Reliability: 14 days for Natal cohort; 7-10, mean 8 days for Manizales cohort ) | Community (including senior centers) | 300 (Natal: 150; Manizales: 150) | Natal: 69.6; Manizales: 69.1 | 50.00% | Mobility Assessment Tool (MAT-sf) | Reliability; Convergent validity, Known-groups validity |
| Kim 2018 | USA | Cross-sectional and test-retest | 2 weeks for reliability | outpatients | 66 | 78.4 | 43.90% | Mobility Assessment Tool-short form (MAT-sf) | interpretability (correlation between patient reported and proxy reported) |
| Marsh 2015 | USA | Prospective cohort | 6 months | Community (from a clinical trial) | 248 (n=31 for reliability) | 66.9 | 71.00% | Mobility Assessment Tool for Walking (MAT-W) | Reliability; Convergent validity; Responsiveness (sensitive to change) |
| de Laat 2011 | Netherlands | Cross-sectional | NA | Outpatients and nursing home (10%) | 171 (reliability: n=22) | 65 | 29.00% | Questionnaire Rising and Sitting Down (QR&S) | Reliability; Smallest Detectable Difference; SEM |
| Roorda 2005 | Netherlands | Secondary analysis | NA | Communities (home-dwelling) | 759 | 60.7 | 53.00% | Rising and Sitting Down (QR&S) | Internal consistency; Reliability; Convergent validity |
| Fries 2009 | USA | prospective cohort | NA | Community | 180 | 73 | 66.00% | PROMIS fix version and CAT | Information content and reliability |
| Houck 2020 | USA | Cross-sectional | NA | Community (including assisted living communities) | 45 | 77.1 | 60.00% | PROMIS Physical Function (PF) CAT | Convergent validity |
| Reuben 1992 | USA | Cohort study | NR | Mix population (Community slightly over 50%) | 149 | 80 | 78.00% | Modified Rosow-Breslau scale | Predictive validity |
| Hoeymans 1997 | Netherlands | test-retest reliability of a longitudinal study | 2 weeks | Community (part of a study) | 105 | 80 | 0.00% | Self-reported measures of functional status (adapted from the WHO questionnaire) | Reliability |
| Tager 1998 | USA | test-retest reliability | 47-49 hours | Community (part of a study) | 199 | NR | 49.75% | Nagi items (9), Rosow-Breslau items (2), SPPARCS items (2) | Reliability |
| Bergland 2002 | Norway | Cross-sectional and longitudinal follow-up | 1-year | Community | 307 | 80 | 100.00% | Self-reported walking index (RW-index) | Convergent validity and predictive validity |
| Manty 2007 | Finland | Prospective cohort study and cross-sectional analysis | 2 years （reliability: 2 weeks） | Community | 632 (reliability: n=29) | 78 | 75.00% | Preclinical and manifest mobility limitation | Reliability; Convergent validity; predictive validity |
| Rejeski 2008 | USA | Secondary analysis of four studies (OASIS; ADAPT; REACT; TRAIN) | 6 months to 3 years | Community (trial participants) | 1,379 | 65.94-71.82 | 52.58% | Pepper Assessment Tool for Disability (PAT-D): mobility domain | Internal consistency; Reliability; Convergent validity, Responsiveness (sensitivity to change) |
| Freedman 2011 | USA | Cross-sectional | 2-4 weeks for reliability | Community (including residential care setting 15%) | 326 (reliability: 111) | NR | 56.40% | Self-report physical capacity measures in National Health and Aging Trends Study (NHATS) | Internal consistency; Reliability; Convergent validity |
| Wang 2012 | Taiwan | Cross-sectional and test-retest | 2 weeks for reliability | Community | 70 | 73 | 51.43% | Self-Reported Measure of Function (mobility domain) | Reliability; Known-groups validity |
| Kopec 2017 | Canada | Instrument development | NA (Reliability: 1.5 to 4.5 weeks, mean 18.3 days, SD: 3.4 days) | Community | 1,072 | 66.3 | 55.90% | Self-reported measures of walking, running, and lifting abilities | Reliability; Convergent validity |
| Chen 2018 | USA | Prospective cohort | 2 years | Community | 1,594 | NR | NR | Self-report major mobility disability | Sensitivity/specificity and Change scores |
| Hung 2014 | USA | prospective cross-sectional (consecutive recruit participants) | NA | Community (athletes) | 472 | 67 | 43.50% | Lower Extremity Computerized Adaptive Test (LE-CAT). | Internal consistency; Reliability; Convergent validity |

## Appendix E. GRADE assessment details

Table E1: LLFDI-FC: Overall score (8 studies):

| Property | Pooled Results | Total Sample Size | Risk of Bias | Inconsistency | Imprecision | Indirectness | GRADE |
| --- | --- | --- | --- | --- | --- | --- | --- |
| Internal consistency | 2+ Sufficient (Cronbach alpha: 0.96-0.97) | 212 | No | No | No | No | High |
| Reliability | 3+ Sufficient *see forest plot (overall ICC= 0.91 (0.88-0.94) | 132 | No | No | No | No | High |
| Measurement error | 2+; 1- Sufficient (SEM ranges from 1.59-2.9) | 812 | No | Serious | No | No | Moderate |
| Predictive validity | 5- insufficient | 821 | Very serious | No | No | No | Low |
| Convergent validity | 16+; Sufficient | 247 | No | No | No | Serious | Moderate |
| Known groups validity | 2+  (sufficient) | 205 | Very serious | No | No | No | Low |
| Responsiveness | 1+; 1- (Sufficient; inconsistent) | 750 | Very Serious | Serious | No | No | Very low |

Table E2: LLFDI-FC: upper extremity (6 studies)

| Property | Pooled Results | Total Sample Size | Risk of Bias | Inconsistency | Imprecision | Indirectness | GRADE |
| --- | --- | --- | --- | --- | --- | --- | --- |
| Internal consistency | 2+ Sufficient (Cronbach alpha: 0.86-0.94) | 212 | No | No | No | No | High |
| Reliability | 3+ Sufficient *see forest plot (overall ICC= 0.85 (0.77-0.90) | 132 | No | No | No | No | High |
| Measurement error | 2- Insufficient (SEM ranges from 4.2-5.1) | 382 | No | No | No | No | High |
| Convergent validity | 6+; 2-; Sufficient | 268 | No | No | No | Serious | Moderate |
| Known groups validity | 2+  (sufficient) | 205 | Very serious | No | No | No | Low |
| Responsiveness | 1- (insufficient) | 320 | Very serious | NA | No | No | Low |

Table E3: LLFDI- basic L/E: (8 studies):

| Property | Pooled Results | Total Sample Size | Risk of Bias | Inconsistency | Imprecision | Indirectness | GRADE |
| --- | --- | --- | --- | --- | --- | --- | --- |
| Internal consistency | 2+ Sufficient (Cronbach alpha: 0.94-0.96) | 212 | No | No | No | No | High |
| Reliability | 3+ Sufficient *see forest plot (overall ICC= 0.91 (0.70-0.97) | 132 | No | No | No | No | High |
| Measurement error | 2+; 1- Sufficient (SEM ranges from 1.88-4.4) | 812 | No | Serious | No | No | Moderate |
| Predictive validity | 5- Insufficient | 821 | Very serious | No | No | No | Low |
| Convergent validity | 7+; 1-; Sufficient | 268 | No | Serious | No | Serious | Low |
| Known groups validity | 2+  (Sufficient) | 205 | Very serious | No | No | No | Low |
| Responsiveness | 2- (insufficient) | 750 | Very serious | No | No | No | Low |

Table E4: LLFDI- advanced L/E: (8 studies):

| Property | Pooled Results | Total Sample Size | Risk of Bias | Inconsistency | Imprecision | Indirectness | GRADE |
| --- | --- | --- | --- | --- | --- | --- | --- |
| Internal consistency | 2+ Sufficient (Cronbach alpha: 0.95-0.96) | 212 | No | No | No | No | High |
| Reliability | 3+ Sufficient *see forest plot (overall ICC= 0.91 (0.83-0.96) | 132 | No | No | No | No | High |
| Measurement error | 2+; 1- Sufficient (SEM ranges from 2.60-4.3) | 812 | No | Serious | No | No | Moderate |
| Predictive validity | 5- Insufficient | 821 | Very serious | No | No | No | Low |
| Convergent validity | 4+; 2-; Sufficient | 218 | No | Serious | No | No | Moderate |
| Known groups validity | 2+  (Sufficient) | 205 | Very serious | No | No | No | Low |
| Responsiveness | 1+; 1- (insufficient) | 750 | Very serious | Serious | No | No | Very low |

MCID for small change from Beauchamp 2019: overall score = 2, upper extremity subscale and advance L/E = 4; basic L/E = 3.

Table E5: LEFS (4 studies)

| Property | Pooled Results | Total Sample Size | Risk of Bias | Inconsistency | Imprecision | Indirectness | GRADE |
| --- | --- | --- | --- | --- | --- | --- | --- |
| Internal consistency | 1+ Sufficient (Cronbach alpha: 0.99) | 45 | No | NA | Very serious | Serious | Very low |
| Reliability | 3+ Sufficient *see forest plot (overall ICC= 0.97 (0.91-0.99) | 129 | No | No | No | Serious | Moderate |
| Measurement error | 2+ Sufficient (SEM ranges from 0.97-3.6) | 145 | No | No | No | Serious | Moderate |
| Convergent validity | 9+;4- Sufficient | 143 | No | Serious | No | Serious | Low |
| Known groups validity | 1+  (Sufficient) | 73 | Very serious | NA | Serious | No | Very low |

LEFS: MID=9* from inpatients with mean age 44 (https://doi.org/10.1093/ptj/79.4.371)

Table E6: PDA-current (3 studies)

| Property | Pooled Results | Total Sample Size | Risk of Bias | Inconsistency | Imprecision | Indirectness | GRADE |
| --- | --- | --- | --- | --- | --- | --- | --- |
| Internal consistency | 2+ Sufficient (Cronbach alpha: 0.92-0.94) | 110 | No | No | No | No | High |
| Reliability | 1- Insufficient ICC= 0.65 | 39 | No | NA | Very serious | No | Low |
| Convergent validity | 3+;3- Sufficient | 131 | No | Serious | No | No | Moderate |

Table E7: PDA-Change (2 studies)

| Property | Pooled Results | Total Sample Size | Risk of Bias | Inconsistency | Imprecision | Indirectness | GRADE |
| --- | --- | --- | --- | --- | --- | --- | --- |
| Internal consistency | 2+ Sufficient (Cronbach alpha: 0.77-0.87) | 110 | No | No | No | No | High |
| Reliability | 1- Insufficient ICC= 0.66 | 39 | No | NA | Very serious | No | Low |
| Convergent validity | 3+;3- Sufficient | 131 | No | Serious | No | No | Moderate |

Table E8: Mobility assessment tool (MAT)

| Property | Pooled Results | Total Sample Size | Risk of Bias | Inconsistency | Imprecision | Indirectness | GRADE |
| --- | --- | --- | --- | --- | --- | --- | --- |
| MAT-SF |  |  |  |  |  |  |  |
| Structural validity | 1+ Sufficient (Overall fit of the unidimensional model and item level goodness of fit were acceptable) | 1343 | Very serious | NA | No | No | Low |
| Reliability | 3+ Sufficient *see forest plot (overall ICC= 0.91 (0.81-0.96) | 109 | No | No | No | No | High |
| Convergent validity | 9+; 6-; Sufficient; inconsistent | 1877 | No | Serious | No | No | Moderate |
| Known groups validity | 3+  (Sufficient) | 1877 | Very serious | No | No | No | Low |
| MAT-Walking |  |  |  |  |  |  |  |
| Reliability | 1+ for each sub-score, Sufficient (ICC=0.85-0.89) | 31 | No | NA | Very serious | No | Low |
| Convergent validity | 2+ for total walking distance; 1- for each usual and fast walking speed; Sufficient for total distance; in sufficient for walking speed | 248 | No | Serious | No | No | Moderate |
| Responsiveness | 1+; (insufficient) | 750 | Very serious | No | No | No | Low |

## Appendix F. GRADE Criteria for the review

**Rationale**

We conducted the GRADE assessment following the COSMIN handbook for the systematic review of psychometric properties [1]. We used the cut-offs suggested by the COSMIN handbook for assessing the risk of bias and the imprecision [1]. For assessing indirectness and inconsistency, the cut-offs were set according to the consensus of our review team, as the COSMIN handbook suggests the review team consider the specific review context. For indirectness, we considered the possible impact of population characteristics (age and settings) on the psychometric properties of mobility measures [2-4]. For inconsistency, we used a proportion-based approach to identify the number of studies that met pre-specified hypotheses for good measurement properties, because the statistical parameter or test (e.g., Chi-squared test or I^2^) may not be reliable for assessing inconsistency or heterogeneity in observational studies [5]. We provided the specific criteria and cut-offs in the following.

Indirectness:

**Serious:** at least 50% of studies having lower age limit within 10 years of 60 (i.e., 50) or mix setting

**Very serious:** at least 50% of studies having lower age limits beyond 10 years of 60 (i.e., <50) or mixed settings

Risk of bias:


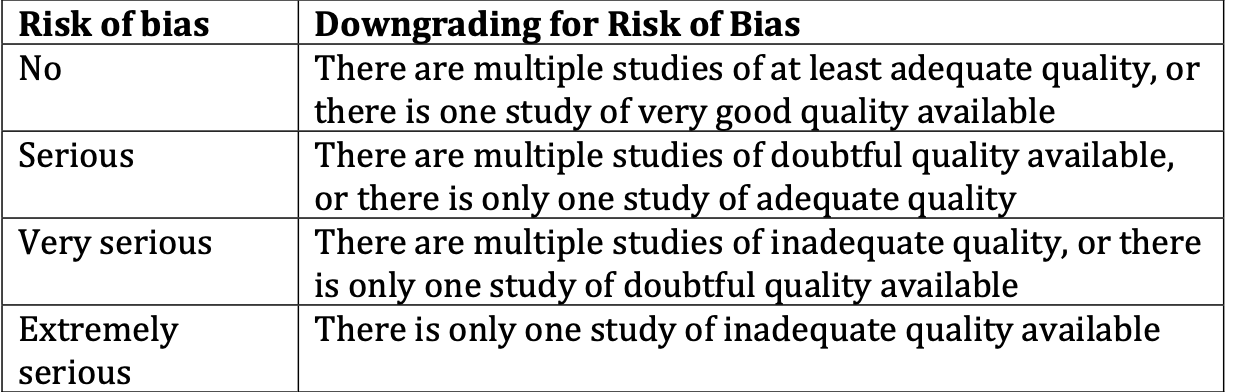


*if there is only 1 study of very good, then no risk of bias

Inconsistency:

**Serious:** if ≥ 50% of results were rated as sufficient according to COSMIN’s criteria for good measurement properties

**Very serious:** if < 50% of results were rated as sufficient according to COSMIN’s criteria for good measurement properties

Imprecision:

**Serious:** if total (pooled/summarized) sample size is between 50 and 100

**Very serious:** if total (pooled/summarized) sample size is less than 50

## Appendix G. Summary of finding tables for internal consistency, measurement error

**Table G1 Internal consistency**

| **Internal Consistency** | **# of tests/hypotheses** | **Summary or pooled result** | **Overall rating** | **Quality of evidence** |
| --- | --- | --- | --- | --- |
| LLFDI-FC: Overall score | 2 | 100% of hypotheses were confirmed; Cronbach alpha: 0.96-0.97 | Sufficient (+) | High |
| LLFDI-FC: upper extremity | 2 | 100% of hypotheses were confirmed; Cronbach alpha: 0.86-0.94 | Sufficient (+) | High |
| LLFDI- basic L/E | 2 | 100% of hypotheses were confirmed; Cronbach alpha: 0.94-0.96 | Sufficient (+) | High |
| LLFDI- advanced L/E | 2 | 100% of hypotheses were confirmed; Cronbach alpha: 0.95-0.96 | Sufficient (+) | High |
| LEFS | 1 | 100% of hypotheses were confirmed; Cronbach alpha: 0.99 | Sufficient (+) | Very low* |
| PDA current | 2 | 100% of hypotheses were confirmed; Cronbach alpha: 0.92-0.94 | Sufficient (+) | High |
| PDA changes | 2 | 100% of hypotheses were confirmed; Cronbach alpha: 0.77-0.87 | Sufficient (+) | High |

*due to serious indirectness and very serious impression.

**Table G2 Measurement error**

| **Measurement Error** | **# of tests/hypotheses** | **Summary or pooled result** | **Overall rating** | **Quality of evidence** |
| --- | --- | --- | --- | --- |
| LLFDI-FC: Overall score | 3 | 66.7% of hypotheses were confirmed; SEM ranges from 1.59-2.9 | Inconsistent (±) | Moderate* |
| LLFDI-FC: upper extremity | 2 | 0% of hypotheses were confirmed; SEM: 4.2-5.1 | Insufficient (-) | High |
| LLFDI- basic L/E | 3 | 66.7% of hypotheses were confirmed; SEM ranges from 1.88-4.4 | Inconsistent (-) | Moderate* |
| LLFDI- advanced L/E | 3 | 66.7% of hypotheses were confirmed; SEM ranges from 2.60-4.3 | Inconsistent (-) | Moderate* |
| LEFS | 2 | 100% of hypotheses were confirmed; SEM ranges from 0.97-3.6 | Sufficient (+) | Moderate& |

SEM: Standard Error of Measurement; *Due to inconsistency; & due to indirectness

**Figure G1: Test-retest reliability for** **Lower extremity functional scale (LEFS)**

**
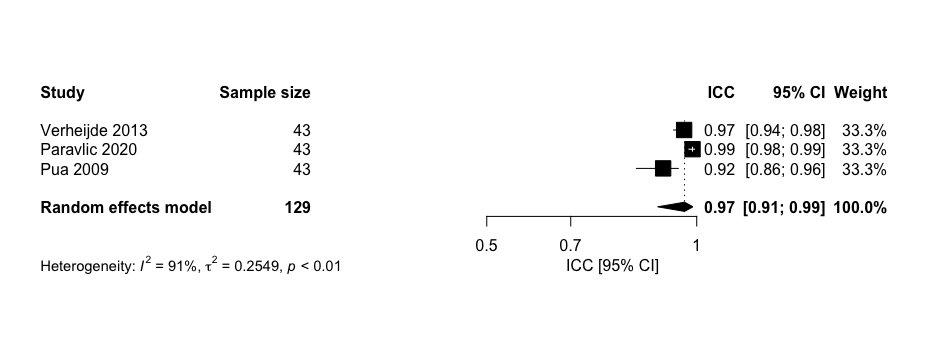
**

**Figure G2: Test-retest reliability for Mobility Assessment Tool (MAT)**


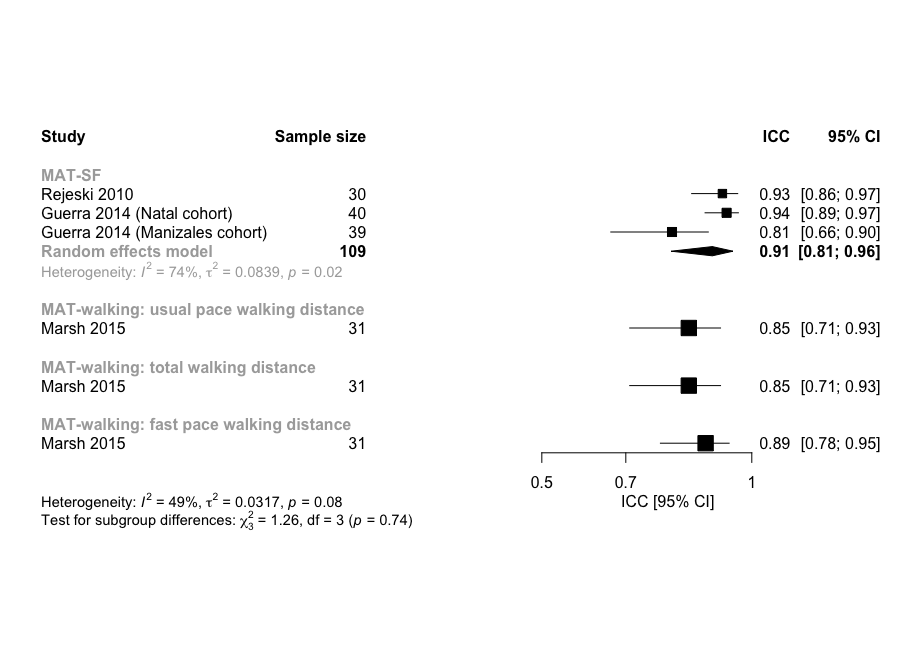


## Appendix H. Reported psychometric property details

**Table H1 Psychometric property details for LLFDI-FC, LEFS, PDA and MAT**

| **Self-reported mobility measures** | **Psychometric property details** |
| --- | --- |
| LLFDI-FC | **Validity**  **Overall score**  The ten-item Physical Functioning Scale of the SF-36 (PF-10) (r = 0.52) [6]; physical component summary (PCS) of the SF-36 (r = 0.67) [6]; RAND-36 physical functioning subscale (r = 0.83) [7]; the London Handicap Scale (LHS) (r = 0.65) [7].  Performance-based measures [7-9] including gait speed, (timed up and go) TUG, the 6-min walk test, timed sit-to-stand, and the Short Physical Performance Battery (SPPB) (absolute r = 0.55 to 0.69).  The LLFDI-FC overall score was also related to the Physical Activity Scale for the Elderly (PASE) (r = 0.56) [7] and Berg Balance Scale (r = 0.48) [9].  **Upper extremity subscale**  The upper extreme subscale showed weak correlations with health-status measures including the PF-10 (r = 0.35) and physical component summary (PCS) of the SF-36 (r = 0.39) [45] and performance-based measures including gait speed (r = 0.26), SPPB (r = 0.19) [8] and TUG (r = -0.34) [9]. However, the correlations between the upper extreme score and Fall Efficacy Scale (FES) (r = -0.57) [10] or Geriatric Depression Scale (GDS) (r= -0.46) [10] were moderate.  **Basic L/E subscale**  The basic lower extreme subscales showed correlations with health-status measures (PF-10 and PCS of the SF-36, r = 0.50 and 0.65) [6]; performance-based measures (gait speed and SPPB, r = 0.66 and 0.63 [8] and TUG, r= -0.49 [9]).  The basic lower extreme subscales showed moderate correlations with FES (r = -0.61), GDS (r = -0.41) [10] and Berg Balance Scale (r = 0.51) [9].  **Advanced L/E**  The advanced lower extreme subscales showed moderate correlations with health-status measures (PF-10 and PCS of the SF-36, r = 0.47 and 0.68) [6] and performance-based measures (gait speed: r = 0.73 [8] and SPPB, r = 0.67 [8] and TUG, r= -0.49 [9]); Berg Balance Scale (r = 0.46) [9]  **Known-group validity**  One study [11] showed that the LLFDI-FC and subscales can distinguish between different levels of functional limitation as measured by the physical functioning subscale (PF-10) of the SF-36. The other study [9] showed that older adults who were cane users had lower overall LLFDI-FC and subscale scores than non-cane users.  **Predictive validity**  One study o reported area under the curve (AUC) values of 0.58 for predicting falls, 0.60 for predicting hospitalization, and 0.67 for low self-reported health [12]. In this study, the odds ratios (ORs) of the LLFDI-FC overall score in predicting emergency department visits was not statistically significant, with an AUC of 0.56. The other study [13] reported the predictive validity for basic and advanced low extremity subscales and showed statistically significant ORs for low self-report health and hospitalizations, while the two subscales did not show statistically significant ORs for falls.  **Responsive**  One study calculated the effect size using the absolute change scores over two years divided by the baseline standard deviation (SD) as well as the effect size in participants who experience a decline or increase in an external anchor (self-related health) [13]. Small or moderate effect sizes were observed for LLFDI-FC overall scale (0.2 to 0.56), basic lower extremity score (0.33 to 0.64), and advanced lower extremity score (0 to 0.54) [13]. The other study reported the Spearman correlation coefficients between changes in the LLFDI-FC and global rating of change, 0.3 for the overall score and advanced lower-extremity score, 0.2 for the upper extremity score and basic lower-extremity score [14].  **Change scores**  Four studies reported the change scores for LLFDI-FC [6, 7, 13, 14]. One study [7] reported the minimum detectable change with 95% confidence (MDC_95_) of 4.3 based on 29 participants and using the ICC from another study [11]. One study [6] reported the absolute and relative (%) smallest real difference (SRD) of 8.0 (14%). Two studies reported the MDC with 90% confidence (MDC_90_) of 3.7 for the overall score [13, 14]. One study [14] reported minimal clinically important difference (MCID) for the small change (overall score: 2; upper-extremity: 4; basic L/E: 3; advanced L/E: 4) and substantial change (overall score: 5; upper-extremity: 10; basic L/E: 6; advanced L/E: 9). |
| **Lower extremity functional scale (LEFS)** | **Validity**  Two studies [15, 16] reported that LEFS had moderate correlations with other functional measures including SF-36 physical function (r = 0.71 and r = 0.75), SF-36 bodily pain (r = 0.60), the linear analog scale of function (r = 0.60), the Western Ontario and McMaster Universities Osteoarthritis Index-physical function (r = 0.78) and performance-based measures including the six-minute walk test, five-meter walk test, gait speed, step test, stair test, and TUG (r = 0.40 to 0.69). One study [16] also reported that LEFS positively associated with the Berg Balance Scale (r = 0.59). Very low-quality evidence from one study [17] supports LEFS had sufficient known group validity.  **Change scores**  One study [16] revealed that LEFS (change/SD = 1.2) is more sensitive to change after physical therapy than SF-36 physical function (change/SD = 0.55), the linear analog scale of function (change/SD = 1.1), Berg Balance Scale (change/SD = 1.0) and performance-based measures (change/SD = 0.62 to 0.90). |
| **Perceived Driving Abilities (PDA) Scale** | **Internal consistency**  Rasch analysis showed that both current and change PDA were unidimensional and hierarchic with good person (reliability index= 0.92 and 0.82) and item (reliability index = 0.96 and 0.90) reliabilities [18].  **Convergent validity**  The current PDA correlated positively with the driving frequency scale (r = 0.46 and 0.51) [18, 19], driving comfort scales during daytime (r = 0.47) or night ( r = 0.59) [18], actual driving behaviour (distance, duration, and other measures) (r = 0.13 to 0.39) [19] and negative correlations with the driving avoidance scale (r = -0.49 or -0.51). The changes in PDA also correlated with driving comfort scales during daytime (r = 0.43) or night ( r = 0.49) [18], the driving frequency scale (r = -0.13) and the driving avoidance scale (r = 0.21) [19].  **Accuracy**  Chen et al. (2021) reported the accuracy of older drivers’ self-awareness of driving ability using PDA against an electronic driving observation schedule based on 108 older drivers. The study reported that the accuracy of the perceived driving ability was 28.7%, with 52.78% of participants overestimating their ability and 18.52% of participants underestimating their ability [20]. |
| **Mobility Assessment Tool (MAT): MAT-short form and MAT-walking** | **Validity**  The MAT-SF correlated with the mobility subscale of the PAT-D [21] (r = -0.60), the PAT-activities of daily living subscale (r = -0.50), and the PAT-D instrumental activities of daily living subscale (r = 0.44). Multiple regression analysis showed the associations between MAT-SF and the 400-metre walk test, SPPB and health status were statistically significant [21-23]. Low-quality evidence showed that MAT-SF had sufficient known group validity (Table 4). Older adults without chronic disease or who can complete the 400-metre walk tests have statistically higher MAT-SF scores than those with comorbidities or who failed the test [21, 23].  One study [24] showed that walking speeds selected for the MAT-W were related to the speed data using the objective measures (GAITRite) [24] for both usual (r = 0.36) and fast walking speeds (r = 0.45). The study also identified the total distance walked from the MAT-W had moderate to high correlations with both a modified version of the Community Health Activities Model Program for Seniors questionnaire (r= 0.66) and minutes of moderate/vigorous physical activity assessed by accelerometry (r = 0.65). |

**Table H2 Reported psychometric properties for other self-reported mobility measures**

| **Self-reported mobility measures** | **Reported psychometric proprietaries** |
| --- | --- |
| **Self-reported walking index (RW-index) [25]** | **Convergent validity:** Timed up-and-go (TUG): Rho = -0.59 (0.56-0.62); SC, stair-climbing height: Rho = 0.57 (0.50-0.64); FR, functional reach: Rho = -0.40 (0.32-0.48); GF, getting up from the floor: Rho = 0.45 (0.33-0.57); CWS, time for walking 29 m at comfortable walking speed: Rho = 0.62 (0.58-0.66), and MWS, at maximal walking speed: Rho = 0.57 (0.54-0.60); FE, walking in a figure of eight: -Rho = 0.59 (0.54-0.64); TS, tandem stance: Rho = 0.52 (0.37-0.67); OS, one-legged stance: Rho = 0.32 (0.21-0.43); FBOS, functional base of support: Rho = 0.29 (0.19-0.39).  **Predictive validity:** RW-index in the worst tertile (sum score 4–9) was a statistically significant predictor of a higher number of falls (p=0.04) among people with two or more falls (odds ratio, OR=1.83, p=0.03, AUC = 0.57); the RW-index associated with the risk of fracture (OR=2.65, p=0.008, AUC=0.61). |
| **Preclinical and manifest mobility limitation [26]** | **Known-groups validity:** participants without mobility limitation had significantly faster walking speeds (11%-20%) and higher muscle power (14%-16%), compared with those with preclinical mobility limitation (P<0.05). Participants with either minor or major manifest limitation had signiﬁcantly slower walking speed (10%-28%), and lower muscle power (13%-26%), compared with participants with preclinical limitation (P<0.05).  **Predictive validity: p**reclinical mobility limitation in the 2-km walk (OR=2.9; 1.2-6.6), 0.5-km walk (OR=1.4; 0.7-2.9) and climb up 1 flight of stairs (OR=1.2; 0.4-3.9) increased the risk for major manifest limitation; Minor manifest limitation in the 2-km walk (OR=8.9; 3.6-21.6), 0.5-km walk (OR=5.4; 2.3-12.2) and climb up 1 flight of stairs (OR=5.4; 1.6-19.1) increased the risk for major manifest limitation. |
| **Pepper Assessment Tool for Disability (PAT-D): mobility domain [27]** | **Internal consistency:** Cronbach’s alphas = 0.87  Test-retest reliability: ICC=0.89  **Convergent validity:** Correlation with WONAC: baseline: Rho=0.65; 6-month: Rho = 0.69; 18-month: Rho=0.73.  Known-groups validity: Faster walkers had better mobility than slow walkers.  **Responsiveness:** The effect size of the mobility subscale was 0.35 after diet plus exercise intervention (average weight loss 5.7%), which driven the changes in the whole scale summary score (effect size: 0.36). |
| **Lower Extremity Computerized Adaptive Test (LE CAT) [28]** | **Internal consistency:** Cronbach’s alphas = 1.0  Reliability: Person Separation index (PSI)= 2.75  **Content validity:** Items demonstrated good fit to the model; average outfit mean square (MNSQ) statistic= 0.79. The average outfit MNSQ for the HOS-ADL was 1.02, the HOS-sports was 0.91, and for the mHHS was 0.92. Unidimensional: unexplained variances of the residuals =1.5%. None of the instruments had any items with disordered thresholds or had item residual correlations greater than 0.8.  **Floor/ceiling effect:** no floor effects; the ceiling effects were acceptable (8.47%). |
| **Modified Rosow-Breslau scale [29]** | **Predictive validity:** Rosow-Breslau scale were not independent predictors of study outcomes in multivariate models (data not displayed). |
| **Self-reported measures of functional status mobility dimension, Nagi items, Rosow-Breslau items, Study of Physical Performance and Age-Related Changes in Sonomans (SPPARCS) items [30]** | **Reliability:** Test-retest agreement= 87%-94%; Weighted Kappa = 0.63-0.74 |
| **Self-report physical capacity measures in National Health and Aging Trends Study [31]** | **Internal consistency:** Cronbach’s alphas: from 0.71 to 0.90  **Reliability:** Kappa: from 0.51 to 0.78; percent agreement: from 82% to 91%  **Convergent validity:** NHATS summary measures of physical capacity associated with Nagi measures and NLTCS activities of daily living summary measure in the expected direction. NHATS items were correlated with frailty, word recall score and mobility score measued by SPPB: Rho= -0.23 to -0.49. |
| **Self-reported measure of function (mobility domain) [32]** | **Reliability:** Kappa= 0.63 |
| **Self-report measures of walking, running and lifting abilities[33]** | **Reliability:** ICC= 0.89 for walking (𝑛 = 287), 0.88 for running (𝑛 = 280), and 0.81 for lifting (𝑛 = 289).  **Convergent validity:** Domains of CAT-5D-QOL: walking summary score: Rho = 0.33 to 0.87, n=1082; running summary score: Rho=0.28 to 0.78, n=1066; lifting summary scoe: Rho=0.29 to 0.52, n=1085; Domain of SF-36: walking summary score: Rho= 0.24 to 0.81, n=550; running summary score: Rho= 0.20 to 0.68, n=535; lifting summary score: Rho= 0.19 to 0.55, n=552.  **Floor/ceiling effects:** Very little ceiling effect; 50.0% found it difficult and 15.4% were unable to run 10 m (floor effect); 76.4% had difficulty running 100 m, 24.5% were unable to run 100 m, and 50.3% were unable to run 1 km; 24.0% stated they could run 10 km. Very few respondents reported problems lifting weights up to 1 kg, but 24.7% had difficulty lifting 4 kg and 51.1% had difficulty lifting 10 kg (3.1% were unable); 75.0% said they could lift 50 kg (7.3% without difficulty). |
| **Self-report major mobility disability [34]** | **Sensitivity/Specificity:** Objective major mobility disability as the gold standard: SR-1/4mail, SR-Blocks, SR-1/4 mile-stairs, SR-blocks-stairs sensitivity was relatively low (from 0.2 to 0.5); the specificity (from 0.90 to 0.96) was very high. |

**References:**

1. Mokkink LB, Prinsen CA, Patrick DL, Alonso J, Bouter LM, Vet HCd, et al. Guideline for Systematic Reviews of Outcome Measurement Instruments. <https://www.cosmin.nl/tools/guideline-conducting-systematic-review-outcome-measures/>. Assessed date: 2022-03-20. 2018.

2. Beauchamp MK, Hao Q, Kuspinar A, D'Amore C, Scime G, Ma J, et al. Reliability and Minimal Detectable Change Values for Performance-Based Measures of Physical Functioning in the Canadian Longitudinal Study on Aging. J Gerontol A Biol Sci Med Sci. 2021 Oct 13;76(11):2030-8.

3. Hao Q, Kuspinar A, Griffith L, D'Amore C, Mayhew AJ, Wolfson C, et al. Measuring physical performance in later life: reliability of protocol variations for common performance-based mobility tests. Aging Clin Exp Res. 2023 May;35(5):1087-96.

4. Scott V, Votova K, Scanlan A, Close J. Multifactorial and functional mobility assessment tools for fall risk among older adults in community, home-support, long-term and acute care settings. Age and Ageing. 2007;36(2):130-9.

5. Iorio A, Spencer FA, Falavigna M, Alba C, Lang E, Burnand B, et al. Use of GRADE for assessment of evidence about prognosis: rating confidence in estimates of event rates in broad categories of patients. BMJ. 2015 Mar 16;350:h870.

6. Roaldsen KS, Halvarsson A, Sarlija B, Franzen E, Stahle A. Self-reported function and disability in late life - cross-cultural adaptation and validation of the Swedish version of the late-life function and disability instrument. Disability & Rehabilitation. 2014;36(10):813-7.

7. Lapier TK. Utility of the late life function and disability instrument as an outcome measure in patients participating in outpatient cardiac rehabilitation: a preliminary study. Physiotherapy Canada. 2012;64(1):53-62.

8. Sayers SP, Jette AM, Haley SM, Heeren TC, Guralnik JM, Fielding RA. Validation of the Late-Life Function and Disability Instrument. Journal of the American Geriatrics Society. 2004;52(9):1554-9.

9. Melzer I, Kurz I, Sarid O, Jette AM. Relationship between self-reported function and disability and balance performance measures in the elderly. Journal of Rehabilitation Research & Development. 2007;44(5):685-91.

10. Feuering R, Vered E, Kushnir T, Jette AM, Melzer I. Differences between self-reported and observed physical functioning in independent older adults. Disability & Rehabilitation. 2014;36(17):1395-401.

11. Haley SM, Jette AM, Coster WJ, Kooyoomjian JT, Levenson S, Heeren T, et al. Late Life Function and Disability Instrument: II. Development and evaluation of the function component. Journals of Gerontology Series A-Biological Sciences & Medical Sciences. 2002;57(4):M217-22.

12. O'Hoski S, Bean JF, Ma J, So HY, Kuspinar A, Richardson J, et al. Physical Function and Frailty for Predicting Adverse Outcomes in Older Primary Care Patients. Archives of Physical Medicine & Rehabilitation. 2020;101(4):592-8.

13. Beauchamp MK, Jette AM, Ward RE, Kurlinski LA, Kiely D, Latham NK, et al. Predictive validity and responsiveness of patient-reported and performance-based measures of function in the Boston RISE study. Journals of Gerontology Series A-Biological Sciences & Medical Sciences. 2015;70(5):616-22.

14. Beauchamp MK, Ward RE, Jette AM, Bean JF. Meaningful Change Estimates for the Late-Life Function and Disability Instrument in Older Adults. Journals of Gerontology Series A-Biological Sciences & Medical Sciences. 2019;74(4):556-9.

15. Pua YH, Cowan SM, Wrigley TV, Bennell KL. The Lower Extremity Functional Scale could be an alternative to the Western Ontario and McMaster Universities Osteoarthritis Index physical function scale. J Clin Epidemiol. 2009 Oct;62(10):1103-11.

16. Verheijde JL, White F, Tompkins J, Dahl P, Hentz JG, Lebec MT, et al. Reliability, validity, and sensitivity to change of the lower extremity functional scale in individuals affected by stroke. Pm & R. 2013;5(12):1019-25.

17. Stratford PW, Kennedy DM, Riddle DL. New study design evaluated the validity of measures to assess change after hip or knee arthroplasty. J Clin Epidemiol. 2009 Mar;62(3):347-52.

18. MacDonald L, Myers AM, Blanchard RA. Correspondence Among Older Drivers' Perceptions, Abilities, and Behaviors. Topics in Geriatric Rehabilitation. 2008;24(3).

19. Blanchard RA, Myers AM. Examination of driving comfort and self-regulatory practices in older adults using in-vehicle devices to assess natural driving patterns. Accid Anal Prev. 2010 Jul;42(4):1213-9.

20. Chen YT, Gelinas I, Mazer B, Myers A, Vrkljan B, Koppel S, et al. Personal and Clinical Factors Associated with Older Drivers' Self-Awareness of Driving Performance. Canadian Journal on Aging. 2021;40(1):82-96.

21. Rejeski WJ, Ip EH, Marsh AP, Barnard RT. Development and validation of a video-animated tool for assessing mobility. Journals of Gerontology Series A-Biological Sciences & Medical Sciences. 2010;65(6):664-71.

22. Guerra RO, Oliveira BS, Alvarado BE, Curcio CL, Rejeski WJ, Marsh AP, et al. Validity and applicability of a video-based animated tool to assess mobility in elderly Latin American populations. Geriatrics & gerontology international. 2014;14(4):864-73.

23. Rejeski WJ, Marsh AP, Anton S, Chen SH, Church T, Gill TM, et al. The MAT-sf: clinical relevance and validity. Journals of Gerontology Series A-Biological Sciences & Medical Sciences. 2013;68(12):1567-74.

24. Marsh AP, Janssen JA, Ip EH, Barnard RT, Ambrosius WT, Brubaker PR, et al. Assessing Walking Activity in Older Adults: Development and Validation of a Novel Computer-Animated Assessment Tool. Journals of Gerontology Series A-Biological Sciences & Medical Sciences. 2015;70(12):1555-61.

25. Bergland A, Jarnlo G, Laake K. Validity of an index of self-reported walking for balance and falls in elderly women. Advances in Physiotherapy. 2002;4(2):65-73.

26. Manty M, Heinonen A, Leinonen R, Tormakangas T, Sakari-Rantala R, Hirvensalo M, et al. Construct and predictive validity of a self-reported measure of preclinical mobility limitation. Archives of Physical Medicine & Rehabilitation. 2007;88(9):1108-13.

27. Rejeski WJ, Ip EH, Marsh AP, Miller ME, Farmer DF. Measuring disability in older adults: the International Classification System of Functioning, Disability and Health (ICF) framework. Geriatrics & gerontology international. 2008;8(1):48-54.

28. Hung M, Hon SD, Cheng C, Franklin JD, Aoki SK, Anderson MB, et al. Psychometric Evaluation of the Lower Extremity Computerized Adaptive Test, the Modified Harris Hip Score, and the Hip Outcome Score. Orthopaedic Journal of Sports Medicine. 2014;2(12):2325967114562191.

29. Reuben DB, Siu AL, Kimpau S. Predictive validity of self-report and performance-based measures of function and health. Journals of Gerontology. 1992;47(4):M106-M10.

30. Tager IB, Swanson A, Satariano WA. Reliability of physical performance and self-reported functional measures in an older population. Journals of Gerontology: Series A: Biological Sciences and Medical Sciences. 1998;53A(4):M295.

31. Freedman VA, Kasper JD, Cornman JC, Agree EM, Bandeen-Roche K, Mor V, et al. Validation of new measures of disability and functioning in the National Health and Aging Trends Study. Journals of Gerontology Series A: Biological Sciences & Medical Sciences. 2011;66A(9):1013-21.

32. Wang C-Y, Ming-Hsia H, Hui-Ya C, Ren-Hau L. Self-Reported Mobility and Instrumental Activities of Daily Living: Test-Retest Reliability and Criterion Validity. Journal of Aging & Physical Activity. 2012;20(2):186-97.

33. Kopec JA, Russell L, Sayre EC, Rahman MM. Self-Reported Ability to Walk, Run, and Lift Objects among Older Canadians. rehabil. 2017;2017:1921740.

34. Chen H, Rejeski WJ, Gill TM, Guralnik J, King AC, Newman A, et al. A Comparison of Self-report Indices of Major Mobility Disability to Failure on the 400-m Walk Test: The LIFE Study. Journals of Gerontology Series A-Biological Sciences & Medical Sciences. 2018;73(4):513-8.
